# Positive mood-related gut microbiota in a long-term closed environment: a multiomics study based on the “Lunar Palace 365” experiment

**DOI:** 10.1186/s40168-023-01506-0

**Published:** 2023-04-24

**Authors:** Zikai Hao, Chen Meng, Leyuan Li, Siyuan Feng, Yinzhen Zhu, Jianlou Yang, Liangzhe Han, Leilei Sun, Weifeng Lv, Daniel Figeys, Hong Liu

**Affiliations:** 1 Institute of Environmental Biology and Life Support Technology, School of Biological Science and Medical Engineering, Beihang University, Beijing, 100083 China; 2 Key Laboratory of Molecular Medicine and Biotherapy, Ministry of Industry and Information Technology, School of Life Science, Beijing Institute of Technology, Beijing, 100081 China; 3 Beijing Institute of Otolaryngology, Department of Otolaryngology, Head and Neck Surgery, Beijing TongRen Hospital, Capital Medical University, Beijing, 100730 China; 4 Department of Biochemistry, Microbiology and Immunology, Ottawa Institute of Systems Biology, Faculty of Medicine, University of Ottawa, Ottawa, K1H 8M5 Canada; 5 State Key Laboratory of Software Development Environment, School of Computer Science and Engineering, Beihang University, Beijing, 100083 China; 6 State Key Laboratory of Virtual Reality Technology and Systems, School of Computer Science and Engineering, Beihang University, Beijing, 100083 China

**Keywords:** Confined built environments, Long-term closed environments, Gut microbiota, Psychobiotics, Multiomics, Microbiota–gut–brain axis, Mood, Depression, Lunar Palace 365

## Abstract

**Background:**

Psychological health risk is one of the most severe and complex risks in manned deep-space exploration and long-term closed environments. Recently, with the in-depth research of the microbiota–gut–brain axis, gut microbiota has been considered a new approach to maintain and improve psychological health. However, the correlation between gut microbiota and psychological changes inside long-term closed environments is still poorly understood. Herein, we used the “Lunar Palace 365” mission, a 1-year-long isolation study in the Lunar Palace 1 (a closed manned Bioregenerative Life Support System facility with excellent performance), to investigate the correlation between gut microbiota and psychological changes, in order to find some new potential psychobiotics to maintain and improve the psychological health of crew members.

**Results:**

We report some altered gut microbiota that were associated with psychological changes in the long-term closed environment. Four potential psychobiotics (*Bacteroides uniformis*, *Roseburia inulinivorans*, *Eubacterium rectale*, and *Faecalibacterium prausnitzii*) were identified. On the basis of metagenomic, metaproteomic, and metabolomic analyses, the four potential psychobiotics improved mood mainly through three pathways related to nervous system functions: first, by fermenting dietary fibers, they may produce short-chain fatty acids, such as butyric and propionic acids; second, they may regulate amino acid metabolism pathways of aspartic acid, glutamic acid, tryptophan, etc. (e.g., converting glutamic acid to gamma–aminobutyric acid; converting tryptophan to serotonin, kynurenic acid, or tryptamine); and third, they may regulate other pathways, such as taurine and cortisol metabolism. Furthermore, the results of animal experiments confirmed the positive regulatory effect and mechanism of these potential psychobiotics on mood.

**Conclusions:**

These observations reveal that gut microbiota contributed to a robust effect on the maintenance and improvement of mental health in a long-term closed environment. Our findings represent a key step towards a better understanding the role of the gut microbiome in mammalian mental health during space flight and provide a basis for future efforts to develop microbiota-based countermeasures that mitigate risks to crew mental health during future long-term human space expeditions on the moon or Mars. This study also provides an essential reference for future applications of psychobiotics to neuropsychiatric treatments.

Video Abstract

**Supplementary Information:**

The online version contains supplementary material available at 10.1186/s40168-023-01506-0.

## Introduction

The space environment, especially the long-term closed isolation environment, will cause the crew members to be in a state of chronic stress for a long time. This could easily induce psychological diseases, adversely affecting crew members’ health, living conditions, and work efficiency, and even lead to mission failure and other serious consequences [1–4]. Recently, more evidence has shown that the microbiota–gut–brain axis plays a key role in regulating brain function, especially in emotional processing and behavior [5–7]. Notably, as the preclinical experiments of psychobiotics (Dinan et al. defined psychobiotics as those living organisms, upon sufficient ingestion produces a health benefit to patients with psychiatric illnesses [8]) continue to show exciting results [9–12], improving and treating neuropsychiatric diseases by regulating gut microbiota composition are gradually becoming a research hotspot. Hence, it is attractive to develop microbiota-based countermeasures that mitigate risks to crew mental health during long-term human space expeditions. However, the correlation between gut microbiota and psychological changes inside long-term closed environments is still poorly understood.

Fortunately, the “Lunar Palace 365” experiment provides a rare opportunity to study the interaction between gut microbiota and moods in healthy people. To be specific, the “Lunar Palace 365” experiment has the following features: (1) it is a 370-day, multicrew, closed experiment conducted in Lunar Palace 1 (LP1). LP1 is a closed and manned Bioregenerative Life Support System (BLSS) facility with excellent performance and having almost no material exchange with the outside world. Therefore, microbial exchange can be largely avoided. (2) The environmental conditions (e.g., temperature and moderate) in LP1 are all in a constant state, and the microbiome within LP1 (such as those on air, water, and material surfaces) is strictly monitored and controlled. Thus, the microbial environment in the system remains relatively stable. (3) During the experiment, the crew members worked, ate, and slept according to a fixed schedule. The crew members’ food sources were the same. In addition, their dietary nutrient levels were calculated rigorously and remained essentially constant. (4) The crew members maintain physical and mental health throughout the experiment [13, 14]. (5) The crew members took regular mood measurement tests and feces samples in the same environment to minimize systematic errors. Therefore, the crew members maintained a long-term stability of their dietary nutrient levels, work and rest times, health status, and exposure to the environmental microbiome. The “Lunar Palace 365” is an ideal experimental platform for studying the relationship between the gut microbiota and mood owing to its isolation from various uncontrollable influences.

In a previous study, we elucidated the patterns of crew members’ psychological changes in a long-term closed isolation environment with the help of the “Lunar Palace 365” experiment [13, 14]. In this study, four potential psychobiotics (*Bacteroides uniformis*, *Roseburia inulinivorans*, *Eubacterium rectale*, and *Faecalibacterium prausnitzii*) were identified through the correlation analysis between gut microbiota and psychological changes. Moreover, based on metagenomic, metaproteomic, and metabolomic analyses, we determined the functional mechanism underlying the effect of these potential psychobiotics on mood. Furthermore, this functional mechanism was analyzed and verified using chronic unpredictable mild stress (CUMS)-induced depression- and anxiety-like rats.

## Results

### Multiomics study based on the “Lunar Palace 365” experiment

**Fig. 1:**
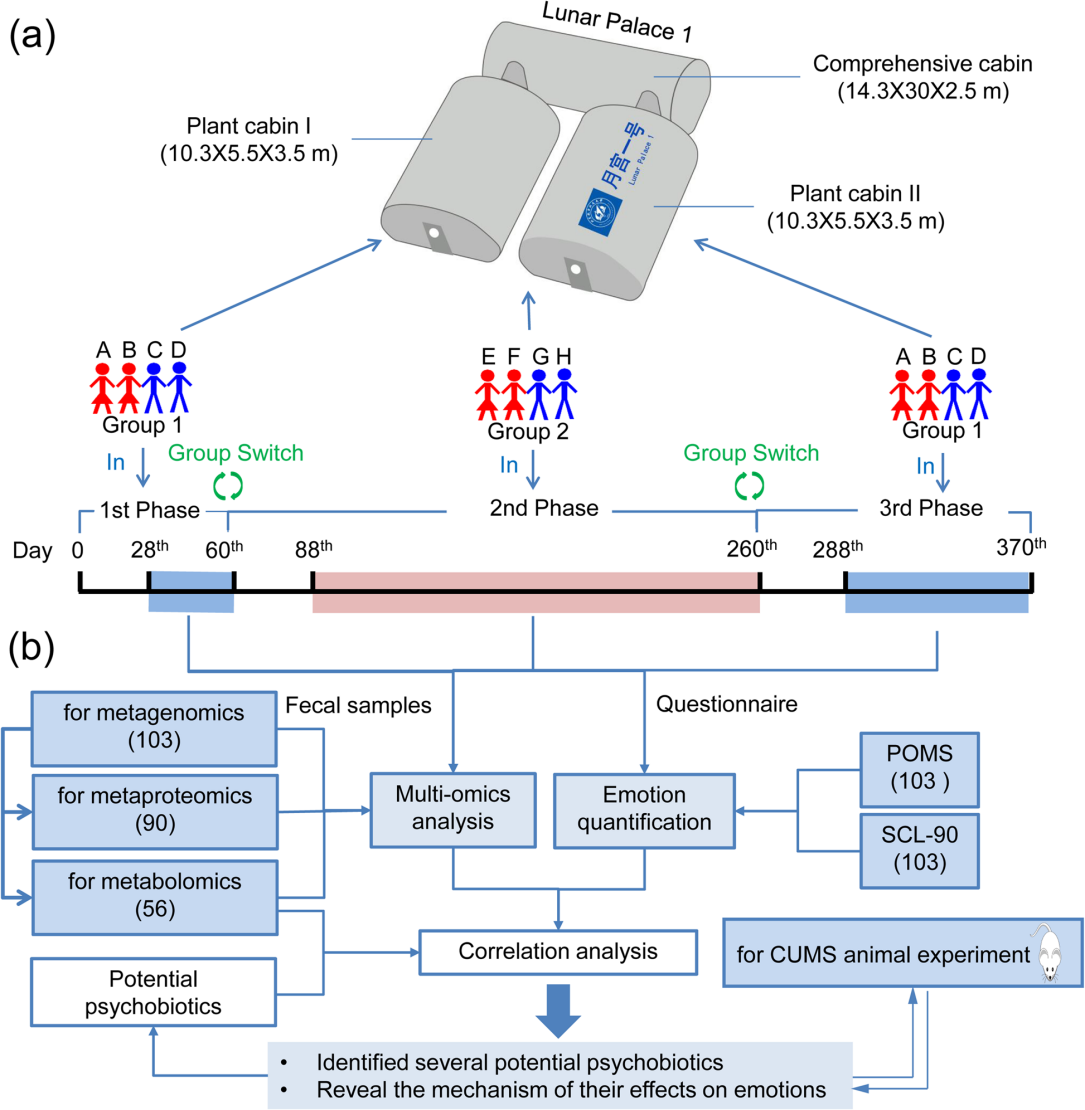
Graphic summary of the study design.** a** Structure of “Lunar Palace 1” and mission arrangement of the “Lunar Palace 365” experiment. **b** Experimental workflow. We collected 103 sets of psychological data and the corresponding fecal samples for correlation analysis. In multiomics analysis, 103, 90, and 56 fecal samples were screened for metagenomic, metaproteome, and metabolomics analyses, respectively. Next, we used CUMS-induced rats to analyze and verify the mechanism and effect of these potential psychobiotics on mood

The “Lunar Palace 365” experiment was conducted in LP1. It comprises two plant cabins and a comprehensive cabin (Fig. 1a). The “Lunar Palace 365” was an experiment of BLSS having the longest isolation time and highest closure coefficient globally and lasted for 370 days from May 10, 2017, to May 15, 2018 [15]. It consisted of three phases with two groups (groups 1 and 2)—the first phase lasted for 60 days (group 1), the second phase lasted for 200 days (group 2), and the third phase lasted for 110 days (group 1, Fig. 1a).

Studies found that it took approximately 21 days for the disturbed gut microbiome to reach a new balance [16, 17]. Considering that the crew members’ diet changed greatly after entering the “LP1” cabin and to avoid the interference of dietary changes on crew members’ gut microbiome as much as possible, we collected crew members’ fecal samples after they had entered the cabin for 28 days for multiomics analysis. Therefore, we collected 103 sets of psychological data and corresponding fecal samples for correlation analysis. In multiomics analysis, 103 fecal samples were screened for metagenomic analysis, 90 fecal samples for metaproteome analysis, and 56 fecal samples for metabolomics analysis. From the correlation analysis between metagenomic profiles of the crew members’ feces and psychological changes, we identified several potential psychobiotics. Thus, on the basis of multiomics analysis analyses, we found the mechanism with which these potential psychobiotics improve mood. Finally, we used CUMS-induced rats to analyze and verify the mechanism and effect of these potential psychobiotics on mood (Fig. 1b).

### Identification of potential psychobiotics

**Fig. 2:**
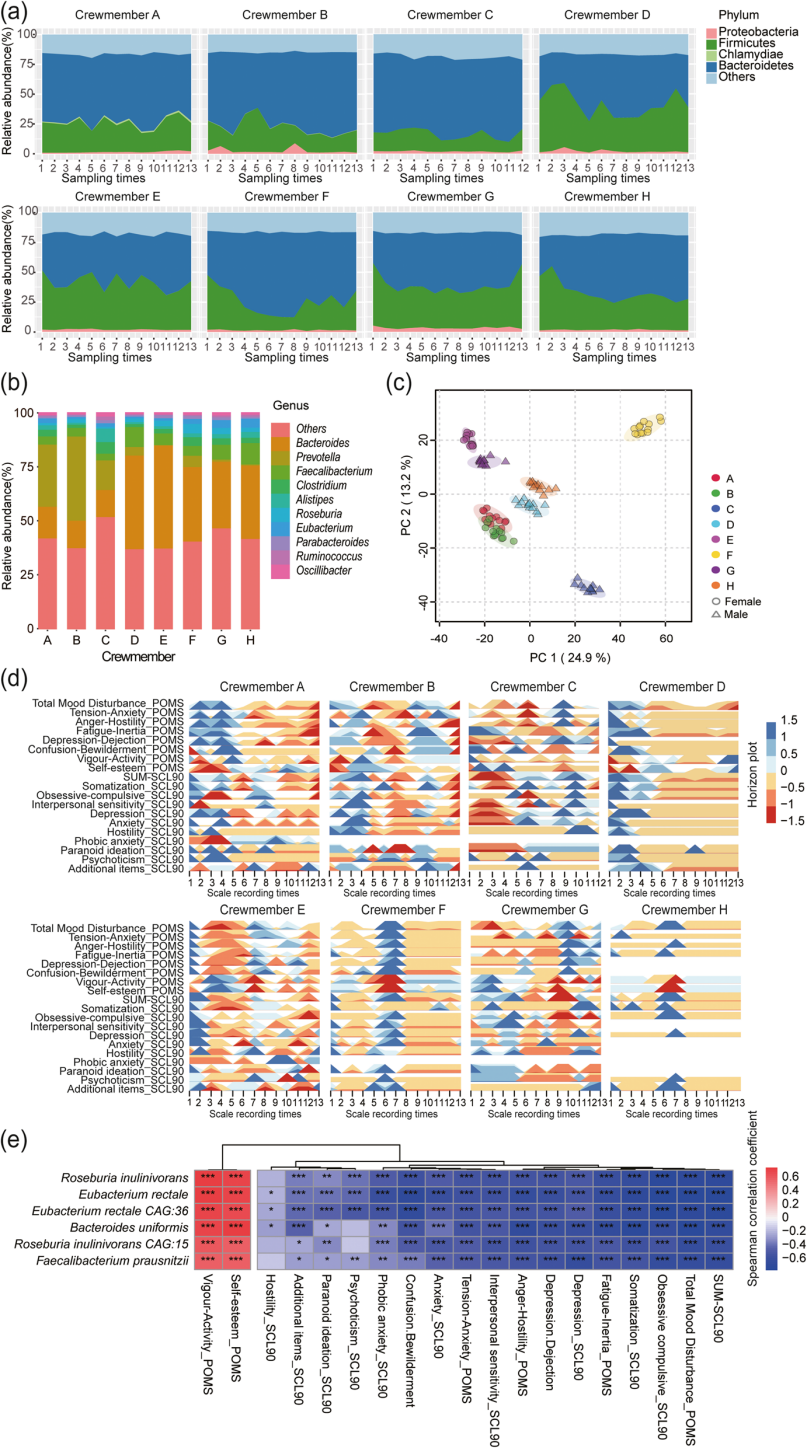
Identification of potential psychobiotics. **a** Percentage stacked graph showing phylum fractional abundances over time. Each ribbon represented a phylum. **b** Top ten genera relative abundance histogram. The top ten genera were selected on the basis of the mean relative abundances of each crew member. The genera relative abundance data of each subject is the average value from all time points. **c** Principal component analysis (PCA) score plots based on the relative abundance of gut microbiota at the individual and gender differences, respectively. **d** Horizon graphs of the variation of the scores of psychological factors over time. Horizon graphs are constructed by the median centering each psychological factor time series. The curve is divided into color bands, and its width is the median absolute deviation. Cooler and warmer regions indicate date ranges where a factor exceeds and below its median score, respectively. The darker the color, the higher the absolute value of the factor score. **e** Heatmap of Spearman’s correlation for potential psychobiotics and psychological factor scores. The potential psychobiotics whose correlation coefficient |R| was ≥ 0.5 (*P* < 0.001) in more than 50% of the psychological factors are shown here. The scaling of correlation coefficient is represented by color depth—a positive correlation is expressed in red and a negative correlation in blue. *P ≤ 0.05, **P ≤ 0.01, ***P ≤ 0.001

To identify potential psychobiotics, we measured and recorded the mood of the crew members on the day of fecal collection. In this study, we collected 103 sets of psychological data and corresponding fecal samples. One-hundred and three fecal samples were sequenced by metagenomics. After quality control, 885,160.47 MbP of clean data (average 8593.79 MbP) was obtained. Then, after gene prediction and redundancy elimination, the proportion of taxonomy annotated to the kingdom level was 88.23%; to the phylum level, 85.92%; to the class level, 81.14%; to the order level was 80.76%; to the family level, 67.80%; to the genus level, 64.61%; and the species level, 43.67%. At the phylum level, on the basis of the composition of gut microbiota of each crew member (Fig. 2a), we found that the dominant phyla were Bacteroidetes, Firmicutes, and Proteobacteria, which accounted for more than 75% of the samples. At the genus level, the top ten genera were selected on the basis of their mean relative abundances to explore the characteristics of gut microbiota of each crew member (Fig. 2b). We found that crew members A, B, and C had *Prevotella* as the dominant microbial genus during the experiment. For crew members D–H, *Bacteroides* was the most abundant genus during the experiment.

Principal component analysis (PCA) was conducted using the relative abundance of gut microbiota at the species level (Fig. 2c; Supplementary Information, Fig. S1) and the scores of psychological factors (Supplementary Information, Fig. S2). Results showed significant differences in the composition of gut microbiota and psychological changes between individual and gender, respectively. To further study whether significant differences corresponded to each individual and gender, we conducted a multivariate analysis of variance (MANOVA) on the basis of the PCA scores of different groups (crew members A–G, female and male). Hierarchical clustering was generated on the basis of Mahalanobis distances due to MANOVA (Supplementary Information, Figs. S1, S2). The MANOVA showed that there were significant differences (*P* < 0.001) between individuals and gender.

Each set of psychological data includes 19 factor scores, including ten factor scores and their total scores (SUM-SCL) of Symptom Checklist-90 (SCL-90), seven factor scores, and the total mood disturbance (TMD) scores of the profile of mood states (POMS) (Fig. 2d). We normalized the scores of psychological factors according to the time series and drew the horizon graphs (Fig. 2d). The results showed that the psychological changes of the crew members were in an unexpected dynamic change with time, and the psychological changes of each crew member had obvious individual and gender differences.

In previous studies, we found that the crew members maintained their mental health during the experiment [13]. To analyze the correlation between gut microbiota and psychological changes, their changes should be a static stochastic process. Under this assumption, we adopted the autocorrelation function (ACF) to test the stationarity of mood scores and intestinal microbial species abundance time series. ACF results (Supplementary Information, Figs. S3 and S4) showed that the change of the psychological score with time and the abundance of the gut microbiota at the phylum level with time were static stochastic processes. Interestingly, we found significant individual and gender differences in the changes of the gut microbiota and mood, and they were all stationary stochastic processes. Thus, we speculate that there may be a correlation between gut microbiota-related and psychological changes. To evaluate the potential presence of such a correlation, the partial least squares (PLS) regression model was used to predict the psychological changes in crew members with the changes in relative abundance of all the gut microbiota species. Results showed that most PLS regression models performed well in correlating the gut microbiota with the psychological factors (Table 1).

**Table 1:** Prediction of psychological factor scores with PLS regression models based on species, KOs, and protein groups

Mood	Metagenomic (species)			Metagenomic (KOs)			Metaproteomic (protein groups)		
	*Q*^2^	*P*	*R*	*Q*^2^	*P*	*R*	*Q*^2^	*P*	*R*
POMS									
Total mood disturbance	0.6861	< 0.0001	0.8403	0.6388	< 0.0001	0.8179	0.5112	< 0.0001	0.7595
Tension–anxiety	0.7718	< 0.0001	0.8490	0.4398	< 0.0001	0.7039	0.6664	< 0.0001	0.7363
Anger–hostility	0.7993	< 0.0001	0.9075	0.4206	< 0.0001	0.7121	0.6918	< 0.0001	0.7782
Fatigue–inertia	0.8901	< 0.0001	0.9350	0.6587	< 0.0001	0.8231	0.7415	< 0.0001	0.8415
Depression–dejection	0.7174	< 0.0001	0.8364	0.5124	< 0.0001	0.7145	0.6369	< 0.0001	0.7545
Confusion–bewilderment	0.9012	< 0.0001	0.8989	0.6554	< 0.0001	0.8369	0.7426	< 0.0001	0.8312
Vigour–activity	0.7896	< 0.0001	0.8800	0.7897	< 0.0001	0.8768	0.8127	< 0.0001	0.9014
Self-esteem	0.8358	< 0.0001	0.9071	0.8221	< 0.0001	0.8888	0.8061	< 0.0001	0.9041
SCL-90									
SUM-SCL90	0.7262	< 0.0001	0.8682	0.7581	< 0.0001	0.8738	0.7225	< 0.0001	0.7800
Somatization	0.7413	< 0.0001	0.8291	0.7524	< 0.0001	0.8335	0.8439	< 0.0001	0.8822
Obsessive–compulsive	0.9495	< 0.0001	0.9534	0.8352	< 0.0001	0.8968	0.7877	< 0.0001	0.8612
Interpersonal sensitivity	0.8949	< 0.0001	0.9166	0.7748	< 0.0001	0.8400	0.8156	< 0.0001	0.8565
Depression	0.8498	< 0.0001	0.9041	0.5676	< 0.0001	0.7735	0.6934	< 0.0001	0.8355
Anxiety	0.8720	< 0.0001	0.9003	0.5757	< 0.0001	0.7749	0.7752	< 0.0001	0.8694
Hostility	0.6883	< 0.0001	0.7702	0.5713	< 0.0001	0.6475	0.8045	< 0.0001	0.8310
Phobic anxiety	0.9487	< 0.0001	0.6590	0.8935	< 0.0001	0.6529	0.8192	< 0.0001	0.6430
Paranoid ideation	0.6615	< 0.0001	0.8227	0.7745	< 0.0001	0.8438	0.7557	< 0.0001	0.8665
Psychoticism	0.8620	< 0.0001	0.7869	0.8162	< 0.0001	0.7041	0.7374	< 0.0001	0.8101
Additional items	0.8619	< 0.0001	0.8627	0.6186	< 0.0001	0.7002	0.8214	< 0.0001	0.8490

*Q*^2^, goodness of prediction based on leave-one-out cross-validated PLS regression. *R,P*, Spearman’s correlation coefficient (R) and *P*-values

The variable importance in projection (VIP) scores in the PLS regression was calculated, and the species with a VIP score of ≥ 1 in more than 50% of the psychological factors were selected as the critical gut microbiota for Spearman’s correlation analysis (Supplementary Information, Table S1). Finally, the species whose correlation coefficient |R|≥ 0.5 (*P* < 0.001) in more than 50% of the psychological factors were shown in Fig. 2e. The results showed that *Bacteroides uniformis*, *Eubacterium rectale*, *Eubacterium rectale CAG:36*, *Roseburia inulinivorans*, *Roseburia inulinivorans CAG15*, and *Faecalibacterium prausnitzii* were significantly positively correlated with the change of positive mood (*P* < 0.05) and negatively associated with the evolution of negative mood (*P* < 0.05). Hence, these strains were further analyzed as potential psychobiotics. We also performed ACF to test the autocorrelation of the potential psychobiotics time series. The results showed that the changes of the potential psychobiotics with time were also a static stochastic process (Supplementary Information, Fig. S5).

### Metagenomic functional analysis of potential psychobiotics

To assess the gut microbial functions, we annotated the 103 metagenomics samples using the KEGG (Kyoto Encyclopedia of Genes and Genomes) database, obtaining 5074 KEGG ortholog group (KO). PCA and MANOVA were conducted using the relative abundance of metagenomic function at the KO level (Supplementary Information, Fig. S6). Results showed that the KO function composition of the intestinal microbiota of crew members also differed significantly between individuals and genders (*P* < 0.001).

**Fig. 3:**
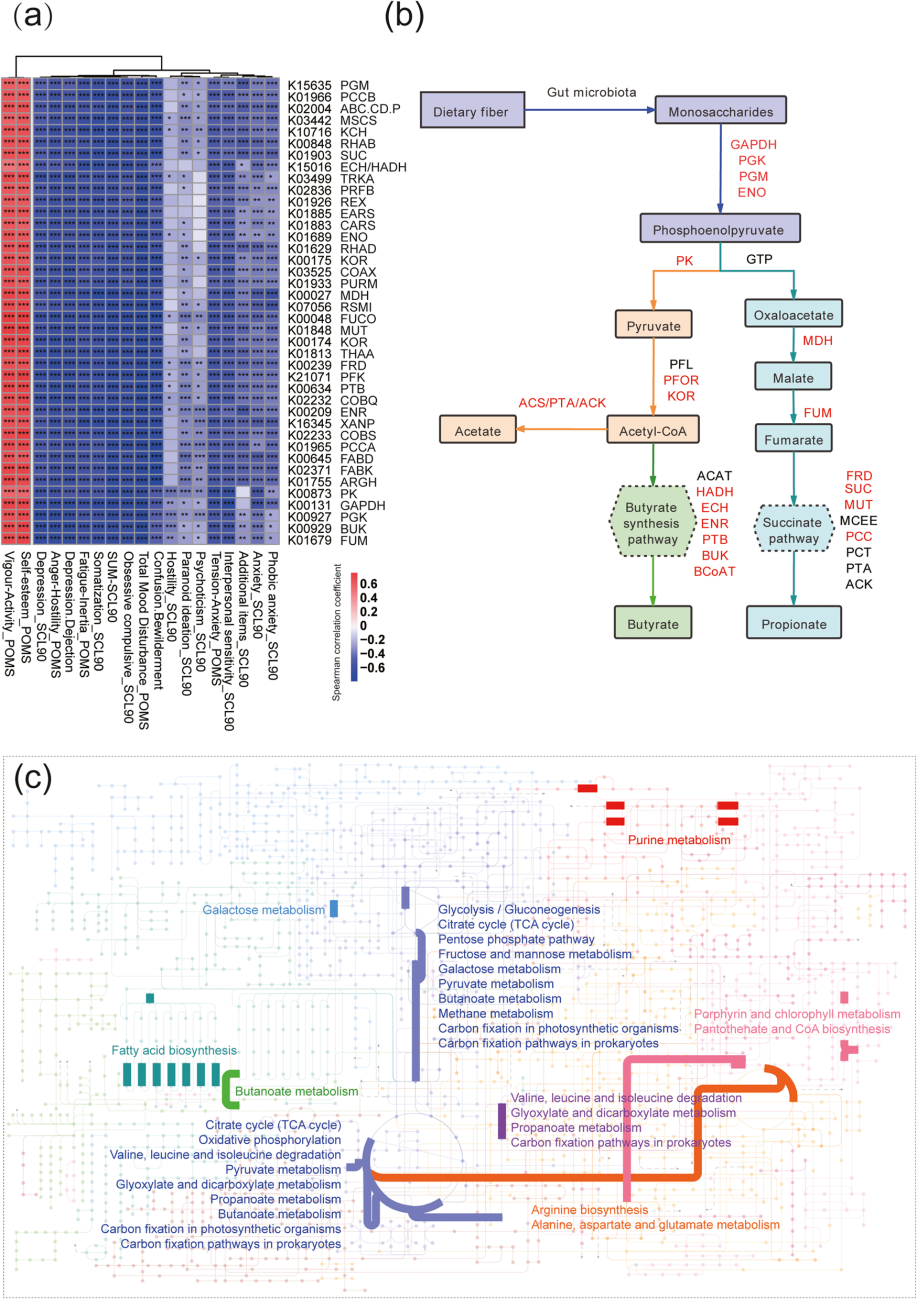
Metagenomic functional analysis of potential psychobiotics.** a** Heatmap of Spearman’s correlation for key Kyoto Encyclopedia of Genes and Genomes ortholog groups (KOs) and psychological factor scores. The KOs whose correlation coefficient |R| was ≥ 0.5 (*P* < 0.001) in more than 50% of the psychological factors are shown here. The scaling of correlation coefficient is represented by color depth—a positive correlation is expressed in red and a negative correlation in blue. *P ≤ 0.05, **P ≤ 0.01, and ***P ≤ 0.001. KOR, 2-oxoglutarate/2-oxoacid ferredoxin oxidoreductase; BUK, butyrate kinase; ENO, enolase; ENR, enoyl-[acyl-carrier protein] reductase; ECH, enoyl-CoA hydratase; HADH, 3-hydroxyacyl-CoA dehydrogenase; FUM, fumarate hydratase; FRD, fumarate reductase; GAPDH, glyceraldehyde-3-phosphate dehydrogenase; MDH, malate dehydrogenase; MUT, methylmalonyl-CoA mutase; PTB, phosphate butyryltransferase; PGK, phosphoglycerate kinase; PGM, phosphoglycerate mutase; PK, pyruvate kinase; SUC, succinyl-CoA synthetase; PCCB, propionyl-CoA carboxylase beta chain; ABC.CD.P, putative ABC transport system permease protein; MSCS, small conductance mechanosensitive channel; KCH, voltage-gated potassium channel; RHAB, rhamnulokinase; TRKA, system potassium uptake protein; PRFB, peptide chain release factor 2; REX, redox-sensing transcriptional repressor; EARS, glutamyl-tRNA synthetase; CARS, cysteinyl-tRNA synthetase; RHAD, rhamnulose-1-phosphate aldolase; COAX, type 3 pantothenate kinase; PURM, phosphoribosylformylglycinamidine cyclo-ligase; RSMI, 16S rRNA (cytidine1402-2′-O)-methyltransferase; FUCO, lactaldehyde reductase; THAA, L-rhamnose isomerase; PFK, ATP-dependent phosphofructokinase/diphosphate-dependent phosphofructokinase; COBQ, adenosylcobyric acid synthase; XANP, xanthine permease; COBS, adenosylcobinamide-GDP ribazoletransferase; PCCA, propionyl-CoA carboxylase alpha chain; FABD, [acyl-carrier-protein] S-malonyltransferase; FABK, enoyl-acyl-carrier protein reductase II; ARGH, argininosuccinate lyase. **b** SCFA-producing metabolic pathways were constructed using the identified KOs. GTP, phosphoenolpyruvate carboxykinase; MCEE, methylmalonyl-CoA epimerase; PCC, propionyl-CoA carboxylase; PCT, propionate CoA transferase; PTA, phosphate acetyltransferase; ACK, acetate kinase; PFL, pyruvate formate-lyase; PFOR, pyruvate, ferredoxin oxidoreductase; ACS, acetyl-CoA synthase; ACK, acetate kinase; ACAT, acetyl-CoA acetyltransferase; BCoAT, butyryl CoA:acetate CoA transferase. **c** Pathways involving these 40 KOs in (**a**), generated using iPATH 3 (https://pathways.embl.de/). These pathways are positively correlated with positive emotions and negatively correlated with negative emotions

To evaluate whether there is a possible correlation between the changes in the KO function of the intestinal microbiota and those in the psychology of the crew members, PLS regression model was used to predict the psychological changes with the changes of all KOs of the gut microbiota, and the results confirmed the high predictability of the model (Table 1). In the PLS regression, the KOs with a VIP score of ≥ 1 in more than 50% of the psychological factors were selected as the key KOs for Spearman’s correlation analysis (Supplementary Information, Table S2). Finally, the KOs whose correlation coefficient |R| was ≥ 0.5 (*P* < 0.001) in more than 50% of the psychological factors are shown in Fig. 3a. We found that 40 KOs were significantly positively correlated with positive mood (*P* < 0.05) and negatively correlated with negative mood (*P* < 0.05) (Fig. 3a). There are 34 enzymes involved in these 40 KOs, among which 18 enzymes are involved in microbial fermentation to produce short-chain fatty acids (SCFAs) (Fig. 3b). These KOs involve the following pathways: pyruvate metabolism, citrate cycle (TCA cycle), fatty acid biosynthesis, fructose and mannose metabolism, galactose metabolism, glycolysis/gluconeogenesis, glyoxylate and dicarboxylate metabolism, methane metabolism, oxidative phosphorylation, pantothenate and CoA biosynthesis, pentose–phosphate pathway, propanoate metabolism, butanoate metabolism, purine metabolism, valine, leucine and isoleucine degradation, alanine, aspartate and glutamate metabolism, and arginine biosynthesis (Fig. 3c).

### Metaproteomic functional analysis of potential psychobiotics

Notably, when the functions predicted from metagenomics analyses are not necessarily expressed, other omics are used to analyze the function of microbial expression. Thus, in this study, high-resolution mass spectrometry (MS) and MetaPro-IQ bioinformatic workflow established by Figeys [18]. Laboratory were used to quantitatively analyze the proteomes of the intestinal microbiota and estimate its functional activity. We identified 41,070 unique peptides and 13,023 protein groups from the 90 fecal samples. Then, the protein groups were filtered with the criteria that the protein groups should be present in ≥ 50% in each of the crew member groups. Finally, 5324 protein groups were selected. PCA and MANOVA were also conducted on the basis of the label-free quantification (LFQ) intensities of protein groups (Supplementary Information, Fig. S7). Results showed that there were significant differences (*P* < 0.001) in the metaproteomic profiles of the crew members’ gut microbiota between individuals and genders. Furthermore, to analyze the metaproteomic function of potential psychobiotics, we obtained 348 protein groups matched to the potential psychobiotics based on a “protein-peptide bridge” method using an in-house code [19].

**Fig. 4:**
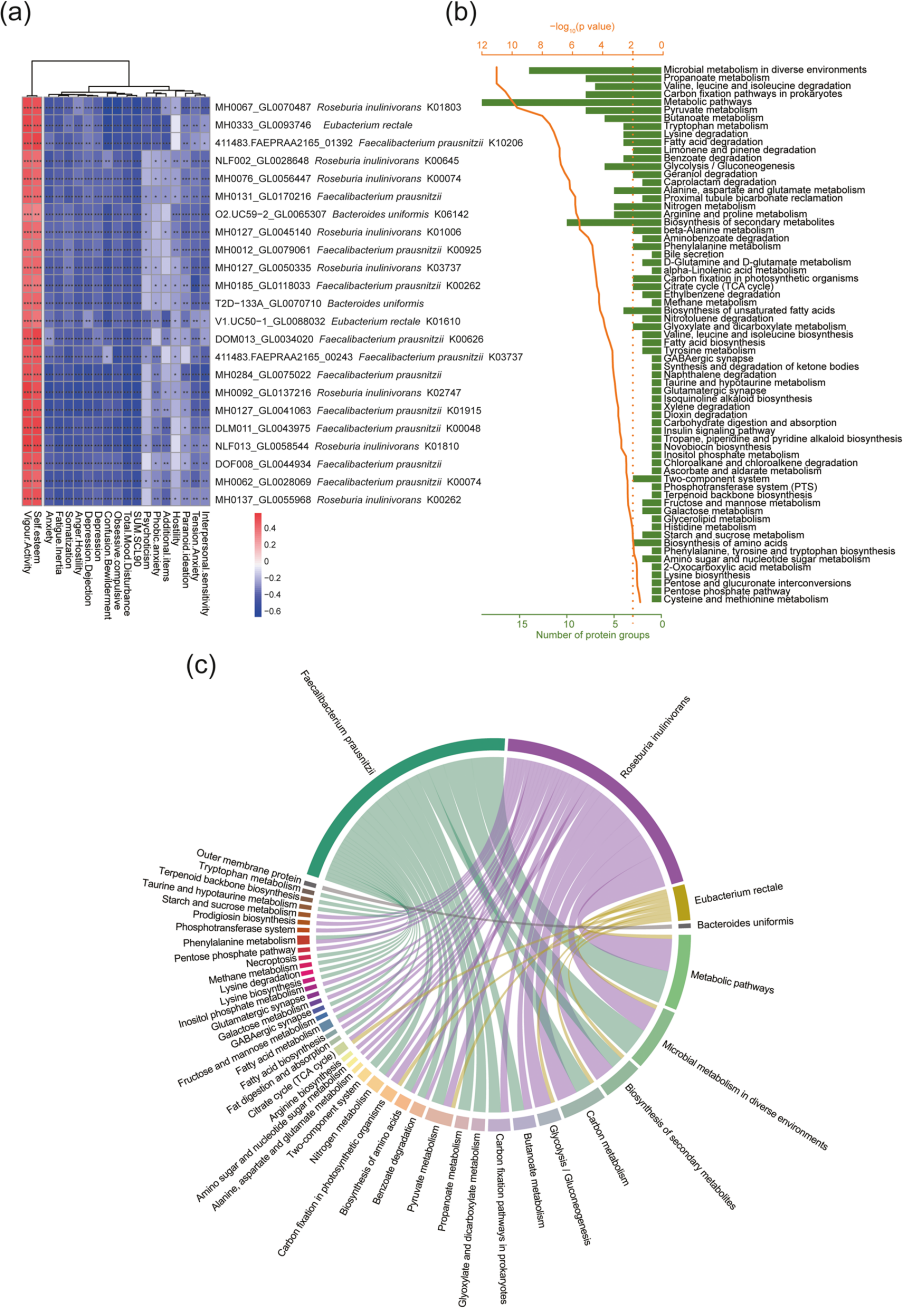
Metaproteomic functional analysis of potential psychobiotics. **a** Heatmap of Spearman’s correlation for key protein groups and psychological factor scores. The protein groups whose correlation coefficient |R| was ≥ 0.35 (*P* < 0.001) in more than 50% of the psychological factors are shown here. The scaling of correlation coefficient is represented by color depth—a positive correlation is expressed in red and a negative correlation in blue. *P ≤ 0.05, **P ≤ 0.01, ***P ≤ 0.001. The taxonomic assignment and KO ID corresponding to each proteome are displayed in (**b**). **b** KEGG functional enrichment analysis based on the key protein groups (*P*-adjusted = 0.05). **c** Taxon-function-coupled enrichment analysis of the key protein groups. Matching taxon and function by using Ultra-deep Metaproteomics established by Daniel Figeys laboratory [19]

To evaluate whether there is a possible correlation between the changes of the 348 protein groups and psychological changes, PLS regression model was used to predict the psychological changes in the crew members with the changes in the 348 protein groups, and the results confirmed the high predictability of the model (Table 1). The protein groups with a VIP score of ≥ 1 in more than 50% of the psychological factors were selected as the key protein groups for Spearman’s correlation analysis (Supplementary Information, Table S3). Finally, the protein groups whose correlation coefficient |R|≥ 0.35 (*P* < 0.001) in more than 50% of the psychological factors were shown in Fig. 4a. Results showed that 23 protein groups were significantly positively correlated with positive moods (*P* < 0.05) and negatively correlated with negative moods (*P* < 0.05) (Fig. 4a). KEGG functional enrichment analysis was conducted using the “enrichment analysis” module on iMetaLab (https://shiny.imetalab.ca/) [20] with these 23 protein groups. Results showed these 23 protein groups involved in 67 KEGG pathways (Fig. 4b). Furthermore, the KO annotation of the 23 protein sequences and taxon-function-coupled analysis was conducted using the GhostKOALA web application [21]. Finally, 18 KOs were obtained (Fig. 4a), and these KEGG pathways were matched with the corresponding potential psychobiotics (Fig. 4c).

### Metabolomic analysis of potential psychobiotics

In the study, 56 fecal samples were collected for nontargeted metabolomics analysis by liquid chromatography (LC)–MS/MS analysis. A total of 3584 chromatographic peaks were identified in positive polarity mode (ES +) and 3335 chromatographic peaks in negative polarity mode (ES −). PCA and MANOVA were also performed on the basis of the fecal metabolites in ES + /ES − (Supplementary Information, Fig. S8). Results showed significant differences (*P* < 0.001) in the composition of fecal metabolites between individuals and genders.

**Fig. 5:**
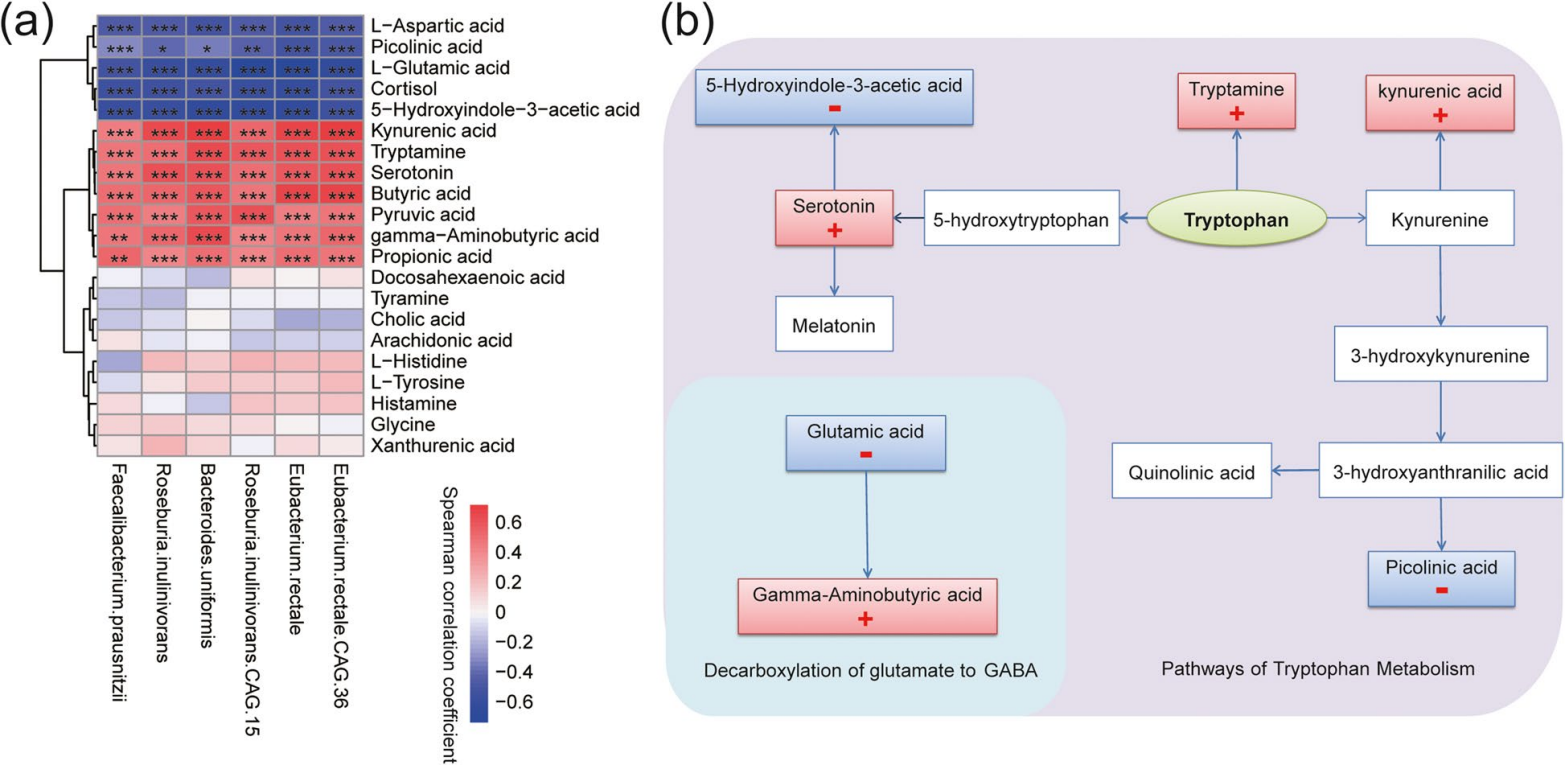
Metabolomic analysis of potential psychobiotics. **a** Heatmap of Spearman’s correlation for key metabolites and potential psychobiotics. The scaling of correlation coefficient is represented by color depth—a positive correlation is expressed in red and a negative correlation in blue. *P ≤ 0.05, **P ≤ 0.01, and ***P ≤ 0.001. **b** Pathways related to the decarboxylation of glucose to gamma–aminobutyric acid (GABA) and tryptophan metabolism as represented by the identified metabolites are significantly associated with potential psychobiotics. “ + ” represents a significant positive correlation with the change in relative abundance of potential psychobiotics, and “ − ” represents a significant negative correlation with the change in relative abundance of potential psychobiotics

To assess whether there were possible associations between the fecal metabolites and potential psychobiotics, PLS regression model was used to predict the relative abundance of potential psychobiotics with the changes in all identified fecal metabolites in ES + /ES − , and the results confirmed the high predictability of the model (Supplementary Information, Table S4). In the PLS regression, the metabolites with a VIP score of ≥ 1 in all potential psychobiotics were selected as the critical metabolites for further analysis (Supplementary Information, Table S5). Then, through literature analysis, we identified 21 nervous system-related metabolites from these key metabolites for Spearman’s correlation analysis (Fig. 5a). Among them, the metabolites significantly related to the changes of potential psychobiotics (*P* < 0.05) were mainly involved in the pathways of decarboxylation of glucose to gamma–aminobutyric acid (GABA) and tryptophan metabolism (Fig. 5b). Additionally, in the tryptophan metabolic pathway, tryptamine, serotonin, and kynurenic acid (KYNA) were significantly positively correlated with the relative abundance of probiotics; however, 5-hydroxyindole-3-acetic acid (5-HIAA) and picolinic acid were significantly negatively correlated with the relative abundance of probiotics (Fig. 5b). In the pathways of decarboxylation of glutamate to GABA, GABA was significantly positively correlated with the relative abundance of pyschobiotics, and glutamate was significantly negatively correlated with the relative abundance of probiotics.

### Effects of potential psychobiotics on the CUMS-induced anxiety-like and depression-like behavior in rats

**Fig. 6:**
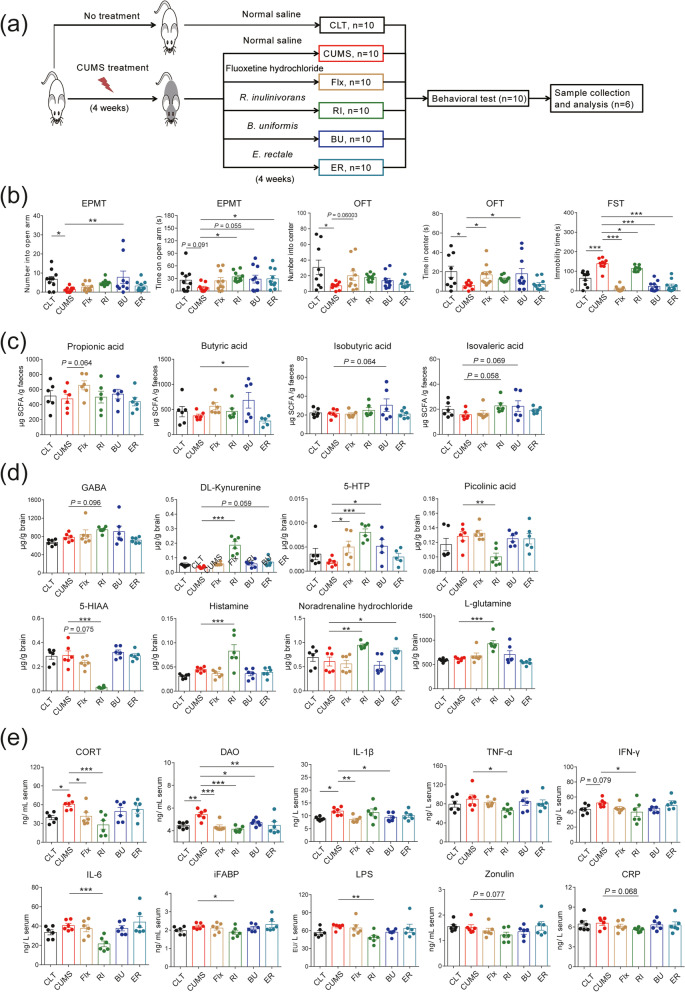
Effects of potential psychobiotics on chronic unpredictable mild stress (CUMS)-induced anxiety-like and depression-like behavior in rats. **a** Rat treatment and experimental procedure. After 4 weeks of CUMS treatment, normal saline (group CUMS), fluoxetine hydrochlorid (group Flx), *B. uniformis* (group BU), *R. inulinivorans* (group RI), and *E. rectale* (group ER) were inoculated into CUMS-induced depression- and anxiety-like rats. After 4 weeks of continuous inoculation, behavioral tests were performed on rats, including elevated plus-maze test (EPMT), open-field test (OFT), and forced swimming test (FST). After euthanasia (carbon dioxide), brain, feces, and blood samples of rats were collected for analysis. **b** Behavioral test results of rats in different groups. EPMT and OFT evaluated the anxiety-like behaviors, and depressive-like behaviors were evaluated by FST. **c** The measurement results of short-chain fatty acids (SCFAs), including propionic acid, butyric acid, isobutyric acid, and isovaleric acid. **d** The measurement results of neurotransmitters, including gamma–aminobutyric acid (GABA), DL-Kynurenine, 5-hydroxytryptophan (5-HTP), picolinic acid, 5-hydroxyindole-3-acetic acid (5-HIAA), histamine, noradrenaline hydrochloride, and L-glutamine. **e** The biochemical measurement results, including corticosterone (CORT), diamine oxidase (DAO), interleukin-1β (IL-1β), tumor necrosis factor-α (TNF-α), interferon-γ (IFN-γ), interleukin-6 (IL-6), intestinal fatty-acid-binding protein (iFABP), lipopolysaccharide (LPS), zonulin, and C-reactive protein (CRP). The values were presented as single data points superimposed to boxplots, and the boxplots were presented with means ± standard error (SE). Differences in the groups were evaluated with one-way ANOVA with Duncan’s test. * *P* ≤ 0.05, ** *P* ≤ 0.01, *** *P* ≤ 0.001

To determine the effect of potential psychobiotics on host mood, *Bacteroides uniformis*, *Roseburia inulinivorans*, *Eubacterium rectale*, and *Faecalibacterium prausnitzii* were inoculated into CUMS-induced depression- and anxiety-like rats, respectively (Fig. 6a). In previous animal experiments, we found that the administration of *F. prausnitzii* led to higher SCFAs and cytokines, interleukin-10 (IL-10), preventing the effects on corticosterone (CORT), C-reaction protein, and interleukin-6 (IL-6) release induced by CUMS. Thus, *F. prausnitzii* had preventive and therapeutic effects on CUMS-induced depression- and anxiety-like behavior [22].

In this study, the behavioral tests showed that, in contrast to the control rats (group CTL), CUMS-treated rats (group CUMS) displayed significantly decreased frequency of entering into the center area (*P* < 0.05). Also, amount of time spent in the center area (*P* < 0.05) in open-field test (OFT) significantly decreased the frequency of entering into open arms (*P* < 0.05) and decreased the amount of time spent in open arms (*P* = 0.091). Furthermore, in elevated plus-maze test (EPMT), it significantly increased amount of immobility time (*P* < 0.001) in forced swimming test (FST) (Fig. 6b), implying that CUMS treatment significantly induced depression- and anxiety-like symptoms. In contrast, fluoxetine hydrochloride (Flx)- and *B. uniformis (BU)*-treated rats displayed a significantly increased amount of time spent in the center area (*P* < 0.05) in OFT; BU-, *R. inulinivorans* (RI)-, and *E. rectale* (ER)-treated rats displayed significantly increased frequency of entering into open arms (*P* < 0.05) or significantly increased amount of time spent in open arms (*P* < 0.05) in EPMT; and Flx-, BU-, RI-, and ER-treated rats displayed significantly decreased amount of immobility time (*P* < 0.05) in FST (Fig. 6b). These results suggest that fluoxetine and potential psychobiotics treatment significantly reduced the CUMS-induced depression- and anxiety-like behavior in rats.

The measurement results of SCFAs show that, compared with CUMS group, propionic acid was increased (*P* = 0.064) in the Flx group; isovaleric acid was increased (*P* = 0.058) in the RI group; butyric acid was significantly increased (*P* < 0.05) in the BU group; and isobutyric acid (*P* = 0.064) and isovaleric acid (*P* = 0.069) were also increased (Fig. 6c). The measurement results of neurotransmitters show that, compared with the CUMS group, 5-hydroxytryptophan (5-HTP) was significantly increased (*P* < 0.05), 5-HIAA was increased (*P* = 0.075) in the Flx group; histamine, L-glutamine, noradrenaline hydrochloride, DL-Kynurenine, and 5-HTP were significantly increased (*P* < 0.05); GABA was increased (*P* = 0.096) in the RI group; picolinic acid and 5-HIAA were significantly decreased (*P* < 0.05) in the RI group; 5-HTP was significantly increased (*P* < 0.05) in the BU group; noradrenaline hydrochloride was significantly increased (*P* < 0.05); and DL-Kynurenine was increased (*P* = 0.059) in the ER group (Fig. 6d).

The biochemical measurement showed that, in contrast to the CTL rats, CORT, interleukin-1β (IL-1β), and diamine oxidase (DAO) in serum of CUMS-treated rats were significantly increased (*P* < 0.05); interferon-γ (IFN-γ) (*P* = 0.079) was increased (Fig. 6e). In contrast to the CUMS rats, CORT, IL-1β, and DAO in serum of Flx-treated rats were significantly decreased (*P* < 0.05); CORT, tumor necrosis factor-α (TNF-α), IFN-γ, IL-6, intestinal fatty-acid-binding protein (iFABP), lipopolysaccharide (LPS), and DAO in serum of RI-treated rats were significantly decreased (*P* < 0.05); zonulin (*P* = 0.077) and C-reactive protein (CRP, *P* = 0.068) were decreased; IL-1β and DAO in serum of BU-treated rats were significantly decreased (*P* < 0.05); and DAO in serum of ER-treated rats was significantly decreased (*P* < 0.05, Fig. 6e).

## Discussion

In this study, we took advantage of the rare opportunity of the “Lunar Palace 365” experiment to study the interaction between gut microbiota and mood of LP 1. This study has unique advantages because it can minimize the influence of other factors (such as diet, lifestyle, and environmental factors) on the gut microbiota besides mood, making it possible to screen potential psychobiotics. On the basis of the multiomics analysis, we found that the species composition, metagenomic functional composition, metaproteome functional composition, and metabolite composition of the gut microbiota of the crew members have significant differences in individual and gender, which was consistent with the previous research results [23, 24]. Furthermore, this difference is probably due to an individual’s unique genetic factors [24–26], life experience, and the interaction between the individual’s gut microbiota as a unique dynamic ecological environment and the surrounding environment [27]. ACF results showed that the changes of the gut microbiota and mood with time had no significant autocorrelation, suggesting stationary stochastic processes. Thus, it is sufficient to study the relationship between gut microbiota and psychological changes.

Furthermore, we found that *Bacteroides uniformis*, *Roseburia inulinivorans*, *Eubacterium recale*, and *Faecalibacterium prausnitzi* were significantly positively correlated with positive moods and negatively correlated with negative moods. Studies have shown that *Bacteroides uniformis* is a potential probiotic, initially isolated from healthy breast-fed infants’ feces [28, 29]. In animal experiments, *B. uniformis* CECT 7771 could induce the production of anti-inflammatory cytokines in vitro and improve the metabolic and immune dysfunction of obese mice induced by a high-fat diet [28, 29]. Genera *Roseburia*, *Eubacterium*, and *Faecalibacterium* are the most abundant bacteria in the human gut microbiota [30], impacting human health using dietary and host-derived polysaccharides and producing the health-promoting metabolite, SCFA, as a fermentation end product [31, 32]. SCFAs are actively involved in the communication of the microbiota–gut–brain axis, which can regulate brain function. For example, it plays a role in the gut–brain axis by regulating the secretion of intestinal hormones (such as GLP-1) [33] and may also directly activate the vagus [34]. SCFAs can modulate immune cell function in the systemic circulation [35] and have direct neuroactive properties [36]. Thus, the positive benefits of SCFA-producing bacteria have made it a novel source of psychobiotics [37].

Additionally, these species can affect colonic motility, immunity maintenance, and anti-inflammatory properties [38–41]. *Roseburia* and *Eubacterium* species are capable of modulating host immunity using the flagella [40]. Metabolites secreted by *Faecalibacterium prausnitzii* are able to block NF-κB activation and IL-8 production [42], which had an anti-inflammatory effect on the host, and then involve the regulation of mood [22]. In previous animal experiments, we found that the administration of *F. prausnitzii* led to an increase in SCFAs and cytokines such as IL-10, inhibiting the inflammatory response induced by CUMS. *F. prausnitzii* can prevent and treat depression- and anxiety-like behavior induced by CUMS [22]. Hence, *F. prausnitzii* has significant potential as a psychobiotic. *E. rectale* is reported to be significantly less present in patients with ulcerative colitis, which implies that *E. rectale* may act as a probiotic [43].

**Fig. 7:**
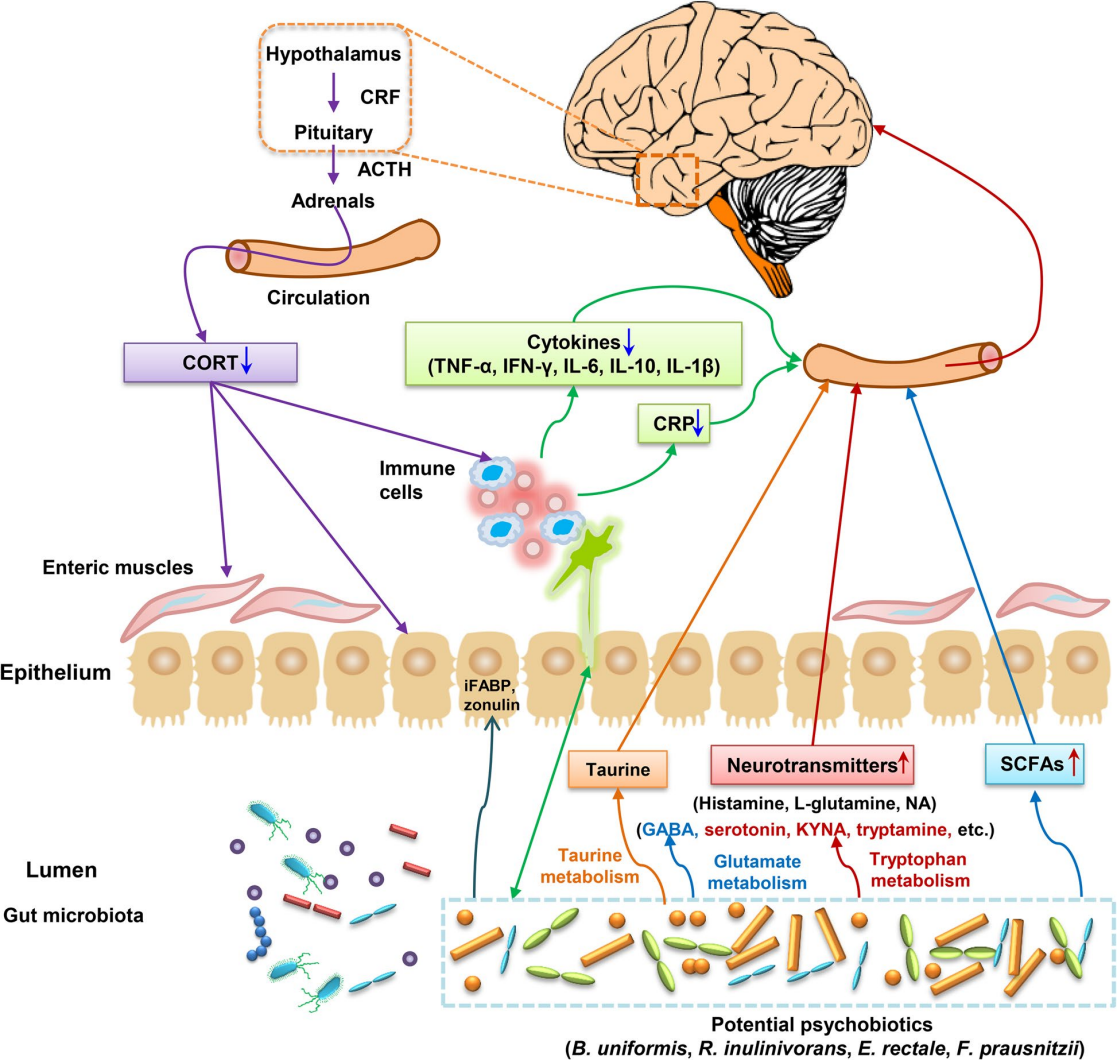
Graphic summary of the main findings. On the basis of the “Lunar Palace 365” experiment and species analysis of metagenomics, we identified four potential psychobiology (*Bacteroides uniformis*, *Roseburia inulinivorans*, *Eubacterium rectale*, and *Faecalibacterium prausnitzii*). Furthermore, on the basis of the multiomics analysis, we determined the functional mechanism of these potential psychobiotics on mood was three pathways related to nervous system functions: first, by fermenting dietary fibers, they may produce short-chain fatty acids such as butyric acid and propionic acid; second, they may regulate amino acid metabolism pathways of aspartic acid, glutamic acid and tryptophan, etc. (e.g., converting glutamic acid to gamma–aminobutyric acid (GABA); converting tryptophan to serotonin, kynurenic acid, or tryptamine); and third, they may regulate other pathways such as taurine metabolism and cortisol metabolism. Furthermore, we verified the effect and mechanism of these potential psychobiotics on mood through animal experiments. The results showed that these potential psychobiotics reduced the CUMS-induced depression- and anxiety-like behavior in rats. Most of the functional mechanisms of these potential psychobiotics on mood found in the “Lunar Palace 365” experiment based on multiomics analysis have been verified in animal experiments. Interestingly, we also found other mechanisms of these potential psychobiotics to improve mood, such as producing other small molecule metabolites (e.g., histamine, L-glutamine and noradrenaline hydrochloride), and reduce the increase in intestinal permeability and inflammatory response caused by CUMS. This improves mood by affecting the enteric nervous system (ENS) and central nervous system (CNS)

On the basis of the KO functional analysis of metagenome and metaproteome, we found many pathways related to polysaccharide metabolism and SCFAs production, which were significantly associated with changes in potential psychobiotics (Fig. 7). These KOs involved glycolysis/gluconeogenesis, carbon fixation pathways in prokaryotes, pyruvate metabolism, citrate cycle (TCA cycle), fructose and mannose metabolism, pentose–phosphate pathway, butanoate metabolism, propanoate metabolism, fatty acid biosynthesis, and other pathways. Notably, 18 enzymes involved in microbial fermentation to produce SCFAs were identified in the metagenomic analysis. In metabolomic analysis, pyruvate, propionic acid, and butyric acid were positively correlated with the relative abundance of these potential pyschobiotics. This indicated that these potential psychobiotics could metabolize polysaccharides (including starch and dietary fiber) to produce SCFAs. The dietary structure strictly in the LP1 followed the pre-designed high-plant and high-fiber diet (Supplementary Information, Table S8). Faith et al. [44]  found that *F. prausnitzi*i strain can utilize potential prebiotic pectin.

On the basis of metagenomic functional analysis, we also found that amino acid-related pathways such as alanine, aspartate, and glutamate metabolism were significantly positively correlated with positive moods. With the help of taxon-function-coupled analysis of metaproteomics, we found that the amino acid biosynthesis, alanine, aspartate, and glutamate metabolism pathways were from *Faecalibacterium prausnitzii* or *Roseburia inulinivorans*. In metabolomic analysis, the changes of neurotransmitters related to the metabolism of aspartic acid, glutamic acid, and tryptophan, such as L-glutamic acid, L-aspartic acid, 5-hydroxyindole-3-acetic acid, and picolinic acid, were significantly negatively correlated with the potential pyschobiotics, while the changes of GABA, tryptamine, serotonin, and KYNA were significantly positively correlated with potential pyschobiotics. Amino acid neurotransmitters, including GABA, glycine, aspartic acid, and glutamic acid, play a notable role in the signal exchange between neurons [45]. Aspartic acid can regulate the metabolism of the brain and nerve [46]. Glutamate, present in over 80% of neurons, is a main excitatory synaptic neurotransmitter [47] and plays a key role in regulating neural plasticity, learning, and memory [48, 49]. However, suppose that the release of glutamate is excessive, in this case, it will produce excitatory neurotoxicity, which has been shown to be associated with many central nervous system (CNS) diseases, including mood disorders and major depression. Most studies have shown that the level of glutamate in patients with mental illness is significantly higher than that in healthy controls [50, 51]. Thus, it has been suggested that reducing the neurotransmission of glutamate may improve mental illness [52, 53]. Glutamate can be converted into GABA, which is a major inhibitory neurotransmitter and plays a crucial role in anxiety and depression disorders in mammals [54]. Recent studies have shown that gut microbiota can modulate gut–brain axis response by producing GABA [55]. In the metabolomic analysis of this study, we found a significant positive correlation between GABA and potential psychobiotics, suggesting that the psychobiotics may convert glutamate to GABA to improve mental state.

Tryptophan is an essential amino acid, which is necessary for the growth and health of animals and humans. More evidence showed that gut microbiota could regulate the neuroendocrine and intestinal immune response by regulating the tryptophan metabolism to produce serotonin, kynurenic acid, tryptamine, indole, and their derivatives [56, 57]. Among them, serotonin is a crucial monoamine neurotransmitter in regulating central nervous transmission and intestinal physiological function [58]. The oxidation of tryptophan produces kynurenine through the kynurenine pathway (KP). KP can clean up the excess tryptophan, affecting the availability of tryptophan. Kynurenic acid (KYNA) not only is an inflammatory mediator but also can cross the blood–brain barrier to reach the CNS; it plays a regulatory role in diverse physiological and pathological processes of brain and gastrointestinal functional disorders [59]. Notably, KYNA is considered to be a neuroprotective N-methyl D-aspartate receptor antagonist [56]. Picolinic acid is synthesized from tryptophan via a sequent side branch of the KP. Thus, similar to other KP metabolites, picolinic acid plays a role in the pathogenesis of inflammatory disorders within the CNS [60]. Tryptamine, a monoamine that is structurally similar to serotonin, can be produced by the decarboxylation of tryptophan-by-tryptophan decarboxylases from bacteria. Tryptamine has been shown to activate trace amine-related receptors expressed in the mammalian brain and regulate the activities of dopaminergic, serotonergic, and glutamatergic systems [61]. 5-HIAA is the primary metabolite of serotonin. Studies have shown that the plasma levels of 5-HIAA were positively correlated with the severity of depression [62]. On the basis of the multiomics analysis, we speculate that potential psychobiotic may regulate the nervous system to improve mood by regulating tryptophan metabolism, such as increasing the production of tryptamine, serotonin, and KYNA and reducing the production of 5-HIAA and picolinic acid.

Additionally, on the basis of the metaproteomic analysis, we found that taurine and hypotaurine metabolism, glutamatergic synapse, and GABAergic synapse from *Faecalibacterium prausnitzii* were significantly positively correlated with positive moods. Studies have shown that taurine is a promising therapeutic tool for treating anxiety-related diseases because it can interact with GABAergic, glycinergic, and glutamatergic receptors [63]. Interestingly, we also found that cortisol was negatively correlated with the change of potential pyschobiotics in metabolomics analysis. Recent studies have shown that gut microbiota could affect the structure and level of cortisol, converting cortisol to androgen [64], or regulating the levels of cortisol and adrenaline in serum by affecting the activity of the hypothalamic–pituitary–adrenal axis (HPA) axis [65]. Thus, we speculated that these potential psychobiotic may improve host mood by regulating taurine and cortisol levels.

To examine the regulatory effect and mechanism of these potential psychobiotics on mood, we conducted therapeutic experiments on CUMS-induced depression- and anxiety-like rats using the potential psychobiotics. The behavioral tests showed that, like the antidepressant drug, fluoxetine, administering the potential psychobiotics, significantly reduced the depression- and anxiety-like behavior of CUMS rats. In contrast to the CUMS rats, we found that the administration of fluoxetine hydrochloride led to higher propionic acid; the administration of *R. inulinivorans* led to higher isovaleric acid; the administration of *B. uniformis* led to higher butyrate, isobutyric acid, and isovaleric acid. In previous animal experiments, we found that administering *F. prausnitzii* led to higher acetic acid, propionic acid, and butyric acid [22]. These potential psychobiotics may produce short-chain fatty acids, which agrees with the functional mechanism of these potential psychobiotics on mood found in the “Lunar Palace 365” experiment based on multiomics analysis. The results of metabolite analysis showed that administering *R. inulinivorans* led to higher GABA, DL-Kynurenine, and 5-hydroxytryptophan and led to lower picolinic acid and 5-HIAA; the administration of *B. uniformis* led to higher 5-hydroxytryptophan; the administration of *E. rectale* led to higher DL-Kynurenine. These metabolite changes involving the metabolism pathways of glutamic acid and tryptophan also agree with the multiomics results in the “Lunar Palace 365” experiment.

We also found that administering *R. inulinivorans* led to higher histamine, L-glutamine, and noradrenaline hydrochloride; the administration of *E. rectale* led to higher noradrenaline hydrochloride. Studies have shown that histamine plays a dual role in nervous system diseases. However, histamine induces a harmful effect by promoting a pro-inflammatory phenotype on microglia; under the attack of lipopolysaccharide, histamine inhibits the injurious effect of microglia-mediated inflammation [66]. Glutamine is intestinal and immune cells’ energy source, which helps maintain the intestinal barrier [67]. Noradrenaline hydrochloride is a neurotransmitter in the catecholamine family, which can mediate various neuroactive functions associated with neurodegenerative diseases [68]. Many important psychiatric drugs have strong effects on the norepinephrine system in the brain, resulting in potentially beneficial or harmful side effects [69]. These results suggest that *R. inulinivorans* and *E. rectale* may affect gut microbiota to produce other small molecule metabolites, such as histamine, L-glutamine, and noradrenaline hydrochloride, which in turn affect the enteric nervous system (ENS) and CNS.

Furthermore, we found that administering *R. inulinivorans* led to lower CORT, TNF-α, IFN-γ, IL-6, iFABP, LPS, DAO, zonulin, and CRP; the administration of *B. uniformis* led to lower IL-1β and DAO; the administration of *E. rectale* led to lower DAO. Thus, the three strains of potential psychobiotics led to lower DAO. Studies have shown that DAO activity in serum was negatively correlated with intestinal permeability of small intestine [70]. In the changes in cytokines and biomarkers caused by *R. inulinivorans*, the decrease in CORT agrees with the results of cortisol metabolism observed by metabolomic analysis in the “Lunar Palace 365” experiment. Zonulin is a physiological regulator of the intercellular tight junction. Increased zonulin levels are accompanied by a leaky intestinal barrier, dysbiosis, and inflammation [71]. The iFABP is located in mature intestinal epithelial cells. When intestinal mucosal damage occurs, iFABP will leak from intestinal epithelial cells into the blood. Hence, iFABP has become a possible noninvasive marker for evaluating gut wall integrity loss and inflammation [72]. Lipopolysaccharide (LPS), the endotoxin of gram-negative bacteria, is now considered a key mediator of a low-grade inflammatory state [73]. TNF-α, IL-1β, and IL-6 is the main inflammatory factor. CRPs are proteins (acute proteins) that rise sharply in plasma when the body is infected or damaged. In a previous study, we found that administering *F. prausnitzii* led to higher cytokine IL-10, preventing the effects on CORT, CRP, and IL-6 release induced by CUMS [22]. Thus, the changes in these immune factors and biomarkers suggest that potential psychobiotics may reduce the increase in intestinal permeability and inflammatory response caused by CUMS and improve mood by affecting ENS and CNS.

## Conclusion

In summary, on the basis of the “Lunar Palace 365” experiment, we analyzed the emotional data and multiomics data of fecal samples, identified four potential psychobiotics, and determined the functional mechanism of these potential psychobiotics on mood. The animal experiments show that these potential psychobiotics reduced the CUMS-induced depression- and anxiety-like behavior in rats. Furthermore, most functional mechanisms of these potential psychobiotics on mood found in the “Lunar Palace 365” experiment based on multiomics analysis have been verified in animal experiments. For example, increasing SCFAs, regulating amino acid metabolism pathways, such as glutamic acid and tryptophan, and regulating cortisol metabolism pathways have been verified in our animal experiments. Additionally, we found other mechanisms of these potential psychobiotics to improve mood, such as producing other small molecule metabolites (e.g., histamine, L-glutamine, and noradrenaline hydrochloride) and reduce the increase in intestinal permeability and inflammatory response caused by CUMS, which in turn improve mood by affecting ENS and CNS. Our findings provide a basis for future efforts to develop microbiota-based countermeasures that mitigate risks to crew mental health during future long-term human space expeditions on the moon or Mars. This study also provides an essential reference for future applications of psychobiotics to neuropsychiatric treatments.

## Materials and methods

### Experimental subjects and design of the “Lunar Palace 365”

The “Lunar Palace 365” experiment was conducted in the Lunar Palace 1 (LP1) of Beihang University. LP1 is a closed operated BLSS facility with excellent performance. Substances necessary for human life, such as oxygen, water, and food, can be recycled in the LP1 to provide life support similar to the earth’s ecological environment. LP1 was designed to contain two plant cabins (plant cabin I and II) and one comprehensive cabin (Fig. 1a). Each plant cabin was divided into two parts with independent environmental condition control. The plant cabin was critical to the regeneration of O^2^ and water as well as food production. The comprehensive cabin of the LP1 included an insect room, washing room, personnel communication, working room, and four bedrooms. The light in the living room (personnel communication and working room and bedroom) of the comprehensive cabin was turned on at 6:30 a.m and turned off at 10:30 p.m. The LED warm white light was used in the living environment. More than 35 kinds of crops were planted in plant cabins, including wheat, potato, soybean, avocado, tomato, strawberry, and various leafy vegetables. The whole BLSS system was closed and completely airtight without any material interaction with the outside. The temperature and humidity in plant cabin I during the whole experiment were 22.25 ± 1 °C and 62.09 ± 5.28% and 22.72 ± 0.89 °C and 61.52% ± 5.11% for plant cabin II. Temperature and humidity control of the plant cabin was stable and conformed to typical planting conditions. Temperature and humidity fluctuations of the comprehensive cabin were 24.11 ± 1.36 °C and 49.62 ± 4.96%, respectively. In the BLSS, the plants absorbed CO_2_ from the air and generated O_2_ via photosynthesis, with the O_2_ used in turn for respiration by the crew, animals, and microorganisms. Thus, regeneration of O_2_ was realized in the BLSS system, maintaining the gas balance of the system. Water output of the crew included domestic wastewater and urine. Domestic wastewater was transferred to a membrane biological-activated carbon bioreactor (MBACR) for purification; the urine of the crew went into rotary evaporation to retrieve elemental nitrogen and water, which was subsequently transferred to the MBACR for purification too. The reclaimed water from the MBACR was transferred into plant nutrient solution preparation tank and then disinfected by UV for plant growth. The irrigation water was purified by evaporation and plant transpiration and was transited to liquid water in plant cabin condensate. After MBACR purification and UV disinfection, the water was then ready for direct use of the crew, including drinking water, sanitary water, and domestic water. Therefore, water recycling and regeneration are realized in the BLSS system [15].

The “Lunar Palace 365” experiment included eight crew members. Group 1 included crew members A (female, 25 years old), B (female, 29 years old), C (male, 25 years old), and D (male, 30 years old). Group 2 included crew members E (female, 25 years old), F (female, 25 years old), G (male, 27 years old), and H (male, 26 years old) (Supplementary Information, Table S7). During the “Lunar Palace 365” experiment, crew members worked, rested, physically exercised, and slept at scheduled times. During the “Lunar Palace 365” experiment in the LP1, the crew members’ dietary nutrient levels were calculated rigorously and remained essentially constant. The dietary structure strictly followed the pre-designed high-plant and high-fiber diet (Supplementary Information, Table S8). Crew members’ mood was measured two to three times a week. Meanwhile, fresh fecal samples were collected on the day of crew members’ mood measurement. And the stool collection time was random on that day. The collected samples were put into cryopreservation tubes and transported out of “Lunar Palace 1” through a logistics channel and stored quickly at − 80 °C until analysis. The general physical examination indexes (performed at the 306th Hospital of PLA, Beijing, China) before and after the experiment in each stage were within the normal range (see Supplementary Information Table S6 for physical examination items). This study was approved by the biomedical ethics committee of Beihang University (approval number: BM20180003).

### Mood measurement and fecal samples collection of crew members

The “Lunar Palace 365” experiment included eight crew members. All crew members gave written informed consent. Crew members’ mood was measured two to three times a week. The psychological status of eight crew members was recorded using SCL-90 and POMS questionnaires via computer or cellphone. Meanwhile, 5-g fresh fecal samples were collected on the day of crew members’ mood measurement and stored quickly at − 80 °C until analysis. Then, each crew member’s 12–13 time points with large psychological changes are randomly selected, and the corresponding psychological data and fecal samples at the time point were selected for further analysis. We collected 103 sets of psychological data and corresponding fecal samples.

### Metagenomic analysis of gut microbiome

Metagenomic sequencing of microbial DNA was conducted on the BGI-SEQ 500 platform (350 bp insert and 100 bp read) [74]. The metagenomics processing workflow was performed as published [74]. Briefly, first, the raw data was pretreated by Readfq to get the clean data. Then, SOAPdenovo was used to assemble and analyze the clean data. MetaGeneMark conducted the open reading frame prediction and filtering with the Scaftigs (≥ 500 bp), removed the redundancy, and obtained the final gene catalog (Unigenes) for subsequent analysis. Then, unigenes and sequences of microbiome extracted from NR (version: 2018.01) database of NCBI were compared by DIAMOND software (Blastp, evaluate ≤ 1e^−5^). For the alignment results of each sequence, the comparison results with the smallest value of ≤ minimum evaluation × 10 were selected for subsequent analysis. After filtering, the lowest common ancestor (LCA) algorithm was adopted to take the classification level, the taxonomic level before the emergence of the first branch was used as the species annotation information of each sequence, and the relative abundance and gene number information of each sample at each classification level (kingdom, phylum, class, order, family, genus, and species) were obtained. Compared unigenes with the KEGG database using DIAMOND software (Blastp, evaluate ≤ 1e^−5^). The relative abundance of the KO was determined by adding the relative lot of each KO gene using the reads on each sample map.

### Metaproteomic analysis of gut microbiome

The collected fecal samples were washed twice with PBS solution and centrifuged at 14,000 g, 4 °C for 20 min. The microbial cell precipitates were collected for further proteomic analysis. According to the procedure published by Zhang Xu et al. [18]. Briefly, first, the prepared protein lysis buffer was added to the EP (Eppendorf) tube containing precipitation, and the lysate was ultrasonically treated at 4 °C for 5 min. After microbial cell lysis, the solution was centrifuged at 16,000 g, 4 °C for 20 min, and the supernatant was precipitated overnight in acidified acetone/ethanol buffer solution at − 20 °C. After centrifugation at 16,000 g, 4° C for 20 min, the protein precipitates were collected, washed three times with glacial acetone, and dissolved in 50-mM ammonium bicarbonate (pH = 8) containing 6-M urea. Next, 10-mm dithiothreitol was used to reduce the 50-μg proteins in each sample, and 20-mm iodoacetamide was used for alkylation. Then, 1-μg trypsin was added and shaken at 37 °C for enzymolysis overnight. Then, the digested peptides were desalted by 10-μm C18 and then analyzed by Q Exactive mass spectrometer (Thermo Fisher Scientific, Waltham, MA, USA).

MetaLab software (v.1.0) was conducted to process the MS raw data according to the MetaPro-IQ bioinformatics workflow of peptide/protein identification and quantification. Briefly, the standard metaproteomic data analysis workflow in MetaLab consists of three modules: database construction, peptide recognition/quantification, and taxonomy analysis. The construction of the database was based on the human gut microbiota gene catalog with 9,878,647 sequences. The Andromeda search engine of MaxQuant was used to characterize peptides. Furthermore, the input proteome list was analyzed by taxonomic enrichment analysis through the enrichment module of iMetalab. The KEGG database was used to annotate protein function, and KO annotation of protein sequence was conducted through the GhostKOALA web application. Through MetaLab, taxon and functions were matched.

### Metabolomic analysis of gut microbiome

We collected crew members’ fecal samples for nontarget metabolomics analysis in this study. All following analysis, identification, and quality control were performed by standard procedures in Novogene Inc. Homogenized fecal samples (100 μg) were resuspended with 500-μL 80% methanol and 0.1% formic acid by fully vertexing. After centrifugation at 15,000 g, 4 °C for 10 min, the supernatant was diluted with ultrapure water to the final concentration containing 60% methanol and centrifuged at 15,000 g, 4 °C for 10 min in a centrifuge tube with a filter membrane. Finally, the filtrate was collected for liquid chromatography-tandem mass spectrometry (LC–MS) analysis. Equal volume samples were taken from each sample and mixed as QC samples, and there were seven QC samples in this study. LC–MS/MS analysis was conducted using vanquish UHPLC system and Orbitrap Q Exactive HF-X mass spectrometer (Thermo Fisher Scientific, Waltham, MA, USA). Compound discoverer v.3.0 (CD 3.0, Thermo Fisher) was used to process the raw data files for peak alignment, peak selection, and quantification of each metabolite. The molecular formula is predicted by normalized data according to additive ions, molecular ion peaks, and fragment ions. Then, the peak was matched with the mzCloud and ChemSpider database to obtain accurate qualitative and relative quantitative results.

### Potential psychobiotics inoculation in CUMS-induced rats

Six-week-old (*n* = 60) rats were allowed to adapt to the environment before experimenting in the first week. Then, the rats were randomly divided into two groups: control group rats (CTL, *n* = 10) did not receive any treatment; CUMS treatment rats (*n* = 50) were exposed to chronic unpredictable mild stress for 4 weeks. After CUMS treatment, the rats were randomly divided into two groups: CUMS group rats (CUMS, *n* = 10) were given normal saline by gavage; Flx group rats (Flx, *n* = 10) were given fluoxetine hydrochloride by gavage (10 mg/kg, Solarbio, Tokyo, Japan); RI group rats (RI, *n* = 10) gavaged at a volume of 1-mL *R. inulinivorans* bacterial solution daily (1 × 10^9^ CFU/mL); BU group rats (BU, *n* = 10) gavaged at a volume of 1-mL *B. uniformis* bacterial solution daily (1 × 10^9^ CFU/mL); and ER group rats (ER, *n* = 10) gavaged at a volume of 1-mL *E. rectale* bacterial solution daily (1 × 10^9^ CFU/mL). After 4 weeks of continuous gavage, all rats in each group were subjected to behavioral tests (open-field, elevated plus-maze, and forced swimming tests) and euthanization (carbon dioxide). Then, six rats in each group were randomly selected for sampling. Blood, feces, and brain samples were collected and stored quickly at − 80 °C until analysis. Blood samples were used for biochemical measurements. IFN-γ, IL-1β, LPS, IL-6, CORT, DAO, TNF-α, CRP, iFABP, and zonulin in serum were measured using enzyme-linked immunosorbent assay kits according to the manufacturer’s protocol (Beijing RGB Technology Development Co., Ltd.). Fecal sample was used for SCFA measurement by gas chromatography (GC)–MS (Thermo TRACE 1310-ISQ LT, USA). Brain sample was used for neurotransmitter measurement (including GABA, DL-Kynurenine, 5-HTP, picolinic acid, 5-HIAA, histamine, L-glutamine, and noradrenaline hydrochloride) by ACQUITY ultra-performance liquid chromatography (UPLC)–MS (Waters Corp., Milford, MA, USA). The experimental workflow is summarized as Fig. 6a.

### Statistical analyses

To facilitate statistical analysis, the relative abundance or LFQ intensity data of metagenomics, metaproteomics, and metabolomics were normalized by log2 transformation and quotient transformation (x/mean) before the examination. For multivariate statistical analysis of metaproteome data, only those proteomes with effective LFQ intensity values in over 50% samples were used. To analyze the psychological data of crew members and whether the gut microbiota was affected by the time effect, MATLAB software was used to construct the ACF and obtain the ACF diagram, Q statistic, and adjoint probability. PCA was conducted in MetaboAnalyst v.4.0. To compare the differences between different groups statistically, MANOVA based on PCA scores was used. The results of MANOVA were hierarchical clustering based on the Mahalanobis distance. To evaluate whether there was a possible correlation between the two variable matrices, PLS regression and Spearman correlation were used for modeling analysis. The prediction performance of the PLS model was evaluated by cross-validation, which was expressed by the root mean square error of cross-validation and good prediction (*Q*^2^). The predictability between predicted and observed mood scores was evaluated by the Spearman correlation coefficient (R) and *P*-value. Finally, by calculating the VIP score in PLS regression and selecting the object with a VIP score greater than 1 as the key object of Spearman correlation analysis for the next step, analysis was conducted. Pathway maps were visualized using iPATH 3 (https://pathways.embl.de/) [75]. Stacked column bars and functional enrichment were visualized on iMetaLab.ca. Data preprocessing, PCA, MANOVA, partial least square regression, and Spearman correlation were conducted using MATLAB (v.R2012b, The MathWorks Inc., Natick, MA, USA). The percentage accumulation map, horizon map, hierarchical cluster, and heatmap of gut microbiome were visualized by R language (v.3.5.3). Statistical analyses were performed using Prism v.6.0 software (GraphPad Software, CA, USA) and OriginPro 2018C (Origin Software, MA, USA) in the rat experiments. Histograms and line charts were presented with means ± standard error (SE). Differences between the groups were evaluated with one-way ANOVA with Duncan’s test. *P* < 0.05 was considered to indicate statistically significant differences. Differences were noted as significant at *P < 0.05, **P < 0.01, and ***P < 0.001.

### Additional methods

Other methods are detailed in online supplementary materials and methods.

## Supplementary Information

**Additional file 1**: Supplementary Methods. Mood measurement of crew members. Metagenomic analysis of gut microbiome. Metaproteomic analysis of gut microbiome. Metabolomic analysis of gut microbiome. Potential psychobiotics inoculation in CUMS-induced rats.

**Additional file 2**: **Fig. S1.** The composition of gut microbiota had significant individual and gender differences. (a, c) PCA scores plots based on the relative abundance of gut microbiota at the species level in different individuals and genders. (b, d) Clustering of different groups based on mahalanobis distances calculated using MANOVA, *** *P*< 0.001.

**Additional file 3:** **Fig. S2.** The scores of psychological factors had significant individual and gender differences. (a, c) PCA scores plots based on the scores of psychological factors in different individuals and genders. (b, d) Clustering of different groups based on mahalanobis distances calculated using MANOVA, *** *P*< 0.001.

**Additional file 4:** **Fig. S3.** The sample autocorrelation function plots (ACF) of the scores variation of each psychological factor. Blue lines indicate upper and lower range of confidential region, sample autocorrelation that falls into the confidential region indicate stability of the mood. The results showed no significant autocorrelation and the crewmembers’ psychological variations were stationary stochastic process.

**Additional file 5:** **Fig. S4.** The sample autocorrelation function plots (ACF) of the gut microbiome composition at the phylum level. Blue lines indicate upper and lower range of confidential region, sample autocorrelation that falls into the confidential region indicate stability of the changes of gut microbiota. The results showed no significant autocorrelation and the changes of gut microbiota with time was a static stochastic process.

**Additional file 6:** **Fig. S5.** The sample autocorrelation function plots (ACF) of the potential psychobiotics. Blue lines indicate upper and lower range of confidential region, sample autocorrelation that falls into the confidential region indicate stability of the changes of potential psychobiotics. The results showed no significant autocorrelation and the changes of potential psychobiotics with time was a static stochastic process.

**Additional file 7:** **Fig. S6.** The relative abundance of metagenomic function at the KO level had significant individual and gender differences. (a, c) PCA scores plots based on the relative abundance of metagenomic function at the KO level in different individuals and genders, respectively. (b, d) Clustering of different groups based on mahalanobis distances calculated using MANOVA, ***, *P*< 0.001.

**Additional file 8:** **Fig. S7.** The protein groups of the gut microbiota had significant individual and gender differences. (a, c) PCA scores plots based on the protein groups of the gut microbiota in different individuals and genders, respectively. (b, d) Clustering of different groups based on mahalanobis distances calculated using MANOVA, ***, *P*< 0.001.

**Additional file 9:** **Fig. S8.** The fecal metabolites in ES +/- had significant individual and gender differences. (a, c) PCA scores plots based on the fecal metabolites in ES +/- in different individuals and genders, respectively. (b, d) Clustering of different groups based on mahalanobis distances calculated using MANOVA, *** *P*< 0.001.

**Additional file 10:** **Fig. S9.** Heatmap of Spearman’s correlation for potential psychobiotics and psychological factor scores. The species whose correlation coefficient |R| was ≥ 0.5 (*P* < 0.001) in more than 50% of the psychological factors are shown here. The scaling of correlation coefficient is represented by color depth—a positive correlation is expressed in red and a negative correlation in blue. *P ≤ 0.05, **P ≤ 0.01, ***P ≤ 0.001.

**Additional file 11:** **Fig. S10.** Heatmap of Spearman’s correlation for key Kyoto Encyclopedia of Genes and Genomes ortholog groups (KOs) and psychological factor scores. The KOs whose correlation coefficient |R| was ≥0.5 (*P* < 0.001) in top 100 of the psychological factors are shown here. The scaling of correlation coefficient is represented by color depth—a positive correlation is expressed in red and a negative correlation in blue. *P ≤ 0.05, **P ≤ 0.01, ***P ≤ 0.001.

**Additional file 12: Fig. S11.** Heatmap of Spearman’s correlation for key protein groups and psychological factor scores. The protein groups whose correlation coefficient |R| was ≥0.35 (*P*<0.001) in top 60 of the psychological factors are shown here. The scaling of correlation coefficient is represented by color depth—a positive correlation is expressed in red and a negative correlation in blue. *P ≤ 0.05, **P ≤ 0.01, ***P ≤ 0.001.

**Additional file 13:** **Table S1.** The key species with VIP score ≥ 1 in more than 50% of the psychological factors.

**Additional file 14:** **Table S2.** The key KOs with VIP score ≥ 1 in more than 50% of the psychological factors.

**Additional file 15:** **Table S3.**The key protein groups with VIP score ≥ 1 in more than 50% of the psychological factors.

**Additional file 16:** **Table S4.** Prediction of potential psychobiotics with PLS regression models based on metabolites. (xlsx 12 kb).

**Additional file 17:** **Table S5.**The key metabolites with VIP score ≥ 1 in all the potential psychobiotics.

**Additional file 18:** **Table S6.** Physical examination items for crewmembers before and after experiment.

**Additional file 19:** **Table S7.** Clinical description of the study participants.

**Additional file 20:** **Table S8.** Major nutrients intake during the “Lunar Palace 365” experiment.

## Data Availability

Metagenomic raw sequence data reported in this paper have been deposited (CRA004621) in the Genome Sequence Archive (GSA, http://bigd.big.ac.cn/gsa) in the BIG Data Center, Chinese Academy of Sciences [76]. CRA004621 is the accession number of GSA. All raw data from LC–MS/MS have been deposited to the ProteomeXchange Consortium (http://www.proteomexchange.org) via the PRIDE [77] partner repository (dataset identifiers PXD027661). Other published datasets used in this work have been listed in supplementary information.

## Abbreviations

LP1: Lunar Palace 1
BLSS: Bioregenerative Life Support System
CUMS: Chronic unpredictable mild stress
PCA: Principal component analysis
MANOVA: Multivariate analysis of variance
SCL-90: Symptom Checklist-90
POMS: Profile of mood states
ACF: Autocorrelation function
PLS: Partial least squares
VIP: Variable importance in projection
KEGG: Kyoto Encyclopedia of Genes and Genomes
KO: KEGG ortholog group
SCFAs: Short-chain fatty acids
MS: Mass spectrometry
LFQ: Label-free quantification
ES +: Positive polarity mode
ES −: Negative polarity mode
GABA: Gamma–aminobutyric acid
5-HIAA: 5-Hydroxyindole-3-acetic acid
CORT: Corticosterone
IL-6: Interleukin-6
IL-10: Interleukin-10
OFT: Open-field test
EPMT: Elevated plus-maze test
FST: Forced swimming test
5-HTTP: 5-Hydroxytryptophan
KP: Kynurenine pathway
KYNA: Kynurenic acid
ENS: Enteric nervous system
CNS: Central nervous system
HPA: Hypothalamic–pituitary–adrenal axis
