# Supplementary material for: Positive mood-related gut microbiota in a long-term closed environment: a multiomics study based on the “Lunar Palace 365” experiment
Source: Microbiome. 2023 Apr 24;11:88. doi: 10.1186/s40168-023-01506-0 (PMC10124008; doi:10.1186/s40168-023-01506-0)
Supplement: Supplementary file 2 — Additional file 1: Supplementary Methods. Mood measurement of crew members. Metagenomic analysis of gut microbiome. Metaproteomic analysis of gut microbiome. Metabolomic analysis of gut microbiome. Potential psychobiotics inoculation in CUMS-induced rats. [file 40168_2023_1506_MOESM1_ESM.docx]

**Supplementary Methods**

**Mood measurement of crew members**

The psychological status of eight crew members was recorded using SCL-90 and POMS questionnaires via computer or cellphone. SCL-90 included 90 self-assessment questions, which reflect ten aspects of psychological symptoms: somatization, obsessive–compulsive, interpersonal sensitivity, depression, anxiety, hostility, phobic anxiety, paranoid ideation, psychoticism, and additional items (mainly reflecting sleep and diet). The total score of SCL-90 (SUM-SCL) was the sum of the scores of each factor. This version of POMS, which consisted of 40 items, were combined into seven factors: tension–anxiety, depression–dejection, anger–hostility, confusion–bewilderment, fatigue–inertia, vigor–activity, self-esteem, and TMD. A TMD score is also calculated by summing the five negative mood scores and subtracting the two positive moods (vigor–activity and self-esteem).

**Metagenomic analysis of gut microbiome**

**DNA extraction and sequencing**

DNA from fecal samples was extracted using the phenol/chloroform method. The extract was treated with DNase-free RNase to eliminate RNA contamination. Then, the concentration and quality of DNA were measured using agarose gel electrophoresis and Qubit 3 fluorometry (Thermo Fisher, Waltham, MA, USA). Metagenomic sequencing was conducted on the BGI-SEQ 500 platform (350 bp insert and 100 bp read).

**Sequencing results pretreatment**

Readfq was used to control the raw data for subsequent analysis. Brief steps were as follows: a) remove the raw reeds with 50% low-quality bases (mass < 38) or more than 15 ambiguous bases; b) remove the reads which overlap with the Adapter was more than 15 bp; and c) map the remaining reads to the human genome (hg19) by Soap V2.22, and filter out the reads that may come from the host.

**Metagene assembly and prediction**

SOAP denovo was used to assemble and analyze the clean data. The assembled scaffolds were interrupted from N connection to get Scaftigs without N. Then, all samples’ clean data were compared with each scaffold by Bowtie2.2.4 software to acquire the PE reads not used. PE reads not used of all samples were mixed assembly and then interrupted the mixed assembly scaffolds from N connection to obtain the Scaftigs sequence without N. Then, MetaGeneMark conducted the open reading frame prediction and filtering with the Scaftigs (≥ 500bp), removed the redundancy, and obtained the final gene catalog (Unigenes) for subsequent analysis.

**Species annotation and KEGG function annotation**

Unigenes and sequences of microbiome extracted from NR (version: 2018.01) database of NCBI were compared by DIAMOND software (Blastp, evaluate ≤ 1e^−5^). For the alignment results of each sequence, the comparison results with the smallest value of ≤ minimum evaluation * 10 were selected for subsequent analysis. After filtering, the lowest common ancestor (LCA) algorithm was adopted to take the classification level, the taxonomic level before the emergence of the first branch was used as the species annotation information of each sequence, and the relative abundance and gene number information of each sample at each classification level (kingdom, phylum, class, order, family, genus, and species) were obtained. Compared Unigenes with the KEGG database using diamond software (Blastp, evaluate ≤ 1e^−5^). The relative abundance of the KO was determined by adding the relative lot of each KO gene using the reads on each sample map.

**Metaproteomic analysis of gut microbiome**

**Metaproteomic sample processing and LC-MS/MS analysis**

The collected fecal samples were washed twice with PBS solution, and centrifuged at 14,000 g, 4°C for 20 min. The microbial cell precipitates were collected for further proteomic analysis. According to the procedure published by Zhang Xu *et al*. ^14^. Briefly, first, the prepared protein lysis buffer was added to the EP (Eppendorf) tube containing precipitation, and the lysate was ultrasonically treated at 4°C for 5 min. The protein lysis buffer consisted of 4% (w/v) sodium dodecyl sulfate, 50 mM Tris-HCl buffer containing 8 M urea (pH = 8.0), a Roche PhosSTOP pill, and a Roche complete Mini pill. After microbial cell lysis, the solution was centrifuged at 16,000 g, 4°C for 20 min, and the supernatant was precipitated overnight in acidified acetone/ethanol buffer solution at −20°C. After centrifugation at 16,000 g, 4°C for 20 min, the protein precipitates were collected, washed three times with glacial acetone and dissolved in 50 mM ammonium bicarbonate (pH = 8) containing 6 M urea. Next, 10 mm dithiothreitol was used to reduce the 50 μg proteins in each sample and 20 mm iodoacetamide was used for alkylation. Then, 1 μg trypsin was added and shaken at 37°C for enzymolysis overnight. Then, the digested peptides were desalted by 10-μm C18 and then analyzed by Q Exactive mass spectrometer (Thermo Fisher Scientific, Waltham, Ma, USA). Briefly, each sample was loaded with a peptide equivalent to 2 μg protein, and the peptide was separated on an analytical column with reverse phase beads (1.9 μm; 120 Å pore size; Dr. Maisch GmbH, Ammerbuch, Germany). A gradient elution of 5%–35% acetonitrile (v/v) was then conducted at a 200 nl/min flow rate for 90 min. The instrument method included a full MS scan from 300 to 1800 m/z, followed by an MS/MS scan of the data related to the 12 most intense ions, the dynamic exclusion repeat count was two, and the repetition exclusion duration was 30 s.

**Metaproteomic data processing**

Metalab software (v.1.0) was conducted to process the MS raw data according to the MetaPro-IQ bioinformatics workflow of peptide/protein identification and quantification. Metalab was a fully automated software platform, which can provide integrated data processing flow for metaproteomic. Briefly, the standard metaproteomic data analysis workflow in Metalab consists of three modules: database construction, peptide recognition/quantification, and taxonomy analysis. The construction of the database was based on the human gut microbiota gene catalog with 9,878,647 sequences.

The second module uses the database of sample characteristics to identify and quantify peptides. The Andromeda search engine of MaxQuant was used to characterize peptides. The determined protein list was generated on the basis of the target bait strategy with FDR < 0.01, and the LFQ strength of all samples was obtained by the MaxLFQ algorithm. Furthermore, the input proteome list was analyzed by taxonomic enrichment analysis through the enrichment module of iMetalab. iMetalab was a cloud analysis platform that allowed general users to obtain the abundance of peptides and taxon directly from the raw MS data. In the taxonomic analysis, the LCA was assigned to the recognized peptide and provided the quantitative information of taxon. The KEGG database was used to annotate protein function, and KO annotation of protein sequence was conducted through the GhostKOALA web application. Through Metalab, taxon and functions were matched.

**Metabolomic analysis of gut microbiome**

We collected crew members' fecal samples for nontarget metabolomics analysis in this study. All following analysis, identification, and quality control were performed by standard procedures in Novogene Inc. Homogenized fecal samples (100 μg) were resuspended with 500 μL 80% methanol, and 0.1% formic acid by fully vertexing. After centrifugation at 15,000 g, 4°C for 10 min, the supernatant was diluted with ultrapure water to the final concentration containing 60% methanol, and centrifuged at 15,000 g, 4°C for 10 min in a centrifuge tube with a filter membrane. Finally, the filtrate was collected for LC-MS analysis. Equal volume samples were taken from each sample and mixed as QC samples, and there were seven QC samples in this study.

LC-MS/MS analysis was conducted using vanquish UHPLC system and Orbitrap Q Exactive HF-X mass spectrometer (Thermo Fisher Scientific, Waltham, MA, USA). Briefly, the samples were analyzed by LC-MS/MS with positive-/negative-ion mode electrospray ionization. Firstly, the sample was injected into a Hyperil Gold column (100 × 2.1 mm, 1.9 μm) using a 16-minute linear gradient at a flow rate of 0.2 mL/min. Then, the extracts were gradient-eluted using different mobile phases: 0.1% FA in water and methanol were used for the positive mode; 5-mM ammonium acetate, pH = 9.0 and methanol were used for the negative mode. The Q Exactive HF-X mass spectrometer was operated with 3.2 kV spray voltage, 320 °C capillary temperature, 35 arb sheath gas flow rate, and 10 arb auxiliary gas flow rate. Compound discoverer v.3.0 (CD 3.0, Thermo Fisher) was used to process the raw data files for peak alignment, peak selection, and quantification of each metabolite. The molecular formula is predicted by normalized data according to additive ions, molecular ion peaks, and fragment ions. Then the peak was matched with the mzCloud and ChemSpider database to obtain accurate qualitative and relative quantitative results.

**Potential psychobiotics inoculation in CUMS-induced rats**

**Animals**

Male Sprague–Dawley (SD) specific pathogen-free (SPF) rats were obtained from SPF Biotechnology Co. Ltd. (Beijing, China). All rats were kept at a 12 h light/dark cycle, constant temperature (21–22°C) and humidity (55% ± 5%). All rats received sterile standard food (SFB Biotechnology Co. Ltd., Beijing, China) and tap water. All animal procedures involved in this study were performed strictly according to research guidelines for the care and use of laboratory animals. Additionally, all procedures were performed according to the National Institutes of Health Guide for the care and use of laboratory animals and were approved by the ethics committee of Beihang University (BM20210108). All mice were randomly assigned, and the experimenter was blind to the assignment of the groups and the evaluation of the results. No samples, animals or data, were excluded.

**Bacterial culture**

*Roseburia inulinivorans* (NCIMB 14030), *Bacteroides uniformis* (ATCC 8492), and *Eubacterium rectale* (JCM 17463) were purchased from the National Collections of Industrial, Food, and Marine Bacteria (NCIMB) and cultured at 37°C in an anaerobic workstation using M2GSC medium. After incubation for 48 h, bacterial suspension was taken for gradient dilution (0, 10^2^, 10^3^, 10^4^, 10^5^, 10^6^) and coated with a plate, which was cultured at 37°C in an anaerobic workstation for 48 h before counting. The bacterial suspension was centrifuged at 25°C at 850 g for 15 mins, and the precipitate was washed with normal saline and repeated once. The concentration of the bacterial solution was 1 × 10^9^ CFU/mL.

**CUMS treatment**

Rats were exposed to CUMS to induce anxiety-like and depressive-like behaviors from the second to fifth week: (1) food deprivation for 24 h; (2) water deprivation for 24 h; (3) rotation on a shaker for 1 h; (4) tail pinch for 1 min; (5) forced swimming (cold water) for 5 min; (6) modified light/dark cycle; (7) hot stress at 50°C for 15 min. Two chronic unpredictable mild stresses in each day were randomly assigned. Control group rats were fed according to the routine feeding and not affected by the other groups.

**Behavioral testing**

All rats in each group were randomly selected for the OFT to measure the anxiety-like behaviors. Rats were placed in the center of the open-field arena (100 × 100 cm, 50 lux) and allowed to explore freely for 5 min. The distance and amount of time the rat spent in the center area and the edges of the chamber within 5 min was recorded using the animal behavior test and analysis system (Ethovision, 3.1 version, Noldus, Tracksys, Nottingham, UK). Ten rats in each group were randomly selected for the EPMT to measure the anxiety-like behaviors. The test was conducted in a plus-maze device (1 m high) consisting of two cross-open arms (425 × 120 mm) and two cross-closed arms (425 × 120 × 225mm). At the beginning of the test, a rat was placed in the central area of the maze facing an open arm, and the amount of time the rat spent in the closed and open arms within 5 min was recorded using the animal behavior test and analysis system (Ethovision, 3.1 version, Noldus, Tracksys, Nottingham, UK). The test room was dark and sound-insulated, tracking instrument recognized the mouse central body point. Before the test, rats were acclimatized to the room for 1 h. After the test, the arms were cleaned with alcohol to remove the odor of the previous rat.

All rats in each group were randomly selected for the FST to measure depressive-like behaviors. Rats were placed in a transparent plastic bucket (40 × 70 cm) filled with water (about 25°C) to 30 cm (kept the head above the water). A video camera positioned directly ahead the bucket was used to track the movement of each rat, and the swimming and immobility time within 5 min was recorded using the animal behavior test and analysis system (Ethovision, 3.1 version, Noldus, Tracksys, Nottingham, UK). The water was completely replaced after every trial.

**Biochemical measurements**

IFN-γ, IL-1β, LPS, IL-6, CORT, DAO, TNF-α, CRP, iFABP, and zonulin in serum were measured using enzyme-linked immunosorbent assay kits according to the manufacturer's protocol (Beijing RGB Technology Development Co., Ltd.). Briefly, biotin, antigen working solution was added, incubated for 1 h and washed for five times. Then, avidin horseradish peroxidase was added, incubated for 1 h and washed for five times. Then, add chromogenic reagents A and B. Finally, the termination solution was added to the test sample. We performed three tests per sample and took the mean value. The limit of detection of IFN-γ, IL-1β, LPS, IL-6, CORT, DAO, TNF-α, CRP, iFABP, and zonulin were determined to be 2 ng/L, 0.2 ng/L, 3 EU/L, 2 ng/L, 2 ng/mL, 0.2 ng/ mL, 4 ng/L, 0.3 mg/L, 0.1 ng/mL, and 0.05 ng/mL, respectively.

**SCFA measurement**

Measure an appropriate amount of pure standards of acetic acid, propionic acid, butyric acid, isobutyric acid, valeric acid, isovaleric acid, and caproic acid, and prepare ten mixed standard concentration gradients with ether (0.02 μg/mL, 0.1 μg/mL, 0.5 μg/mL, 2 μg/mL, 10 μg/mL, 25 μg/mL, 50 μg/mL, 100 μg/mL, 250 μg/mL, and 500 μg/mL, respectively). Take an appropriate amount of fecal sample and add 50-μL 15% phosphoric acid, plus 125 μg/mL internal standard (isocaproic acid) solution 100 μL and ether 400 μL, homogenate for 1 min, centrifuge at 4°C, 12000 rpm for 10min, and take the supernatant for gas chromatography (GC)–MS (Thermo TRACE 1310-ISQ LT, USA).GC conditions: HP-INNOWax capillary column (30 m × 0.25 mm ID × 0.25 μm; Agilent) to separate SCFA. The injection volume was 1 μl in a 10:1 split mode, the injection temperature was 250°C, the ion source temperature was 230°C, the transfer line temperature was 250°C, and the quadrupole temperature was 150°C. Temperature gradient increased at 10°C/min from 90°C to 120°C, increased at 5°C/min from 120°C to 150°C, increased at 25°C/min from 150°C to 250°C, and held for 2 min at 250°C. The carrier gas was helium with a flow rate of 1.0 mL/min. MS conditions: electron impact ionization source, SIM scanning mode, electron energy of 70 eV.

**Neurotransmitter measurement**

Take an appropriate amount of 23 neurotransmitter standards [including GABA, DL-Kynurenine, 5-HTP, picolinic acid, 5-HIAA, histamine, L-glutamine, and noradrenaline hydrochloride] and prepare single standard mother liquor with methanol. Measure an appropriate amount of each mother liquor to make a mixed standard, dilute it to the appropriate concentration with 10% formic acid methanol and H_2_O (1:1), and make a working-standard solution. Weigh an appropriate amount of brain sample into a 2-mL EP tube, add 600-μL 10% formic acid methanol solution and H_2_O (1:1) solution, add 50-mg glass beads and put them into a high-throughput tissue grinder, shake at 55Hz for 1 min, and repeat twice. Then 12000 rpm, centrifugation at 4°C for 5min. Take the supernatant 100 μL, add 100-μL double isotope internal standard with a concentration of 100 ppb, vortex oscillation for 30 s, and the supernatant was at 0.22-μM membrane filtration The filtered solution is added to the detection bottle (detection of low-content substances, including DL-Kynurenine, 5-HTP, picolinic acid, 5-HIAA, histamine, and noradrenaline hydrochloride). Take the original supernatant 20 μL, add 980-μL 10% formic acid methanol solution- H_2_O (1:1) solution, and vortex oscillation for 30 s. Take 100-μL diluted samples，add-100 μL double isotope internal standard with a concentration of 100 ppb, vortex oscillation for 30 s. Supernatant at 0.22-μM membrane filtration, and the filtered liquid was added to the detection bottle (detection of high content substances, including GABA and L-glutamine) for LC–MS online detection.

LC conditions: samples were analyzed at 40°C by high-performance LC on an Acquity UPLC BEH C18 column (2.1 × 100 mm, 1.7 μm; Waters). The injection volume was 5 μl. Mobile phases consisted of (A)10% methanol-water (containing 0.1% formic acid) and (B)50% methanol-water (containing 0.1% formic acid). Solvent B increased from 20% to 100% between 0 and 1 min; solvent B remained at 100% between 1 and 7 min,;solvent B was reduced from 100% to 20% between 7 and 7.5 min; solvent B remained at 20% between 7.5 and 11 min. The flow rate was set as 0.4 mL /min. MS conditions: electrospray ionization source, positive-ion ionization mode. The temperature of the ion source was set at 500°C . The ion source voltage was 5000V. Multiple response monitoring was used for scanning.
