# Supplementary figures and images for: Positive mood-related gut microbiota in a long-term closed environment: a multiomics study based on the “Lunar Palace 365” experiment
Source: Microbiome. 2023 Apr 24;11:88. doi: 10.1186/s40168-023-01506-0 (PMC10124008; doi:10.1186/s40168-023-01506-0)

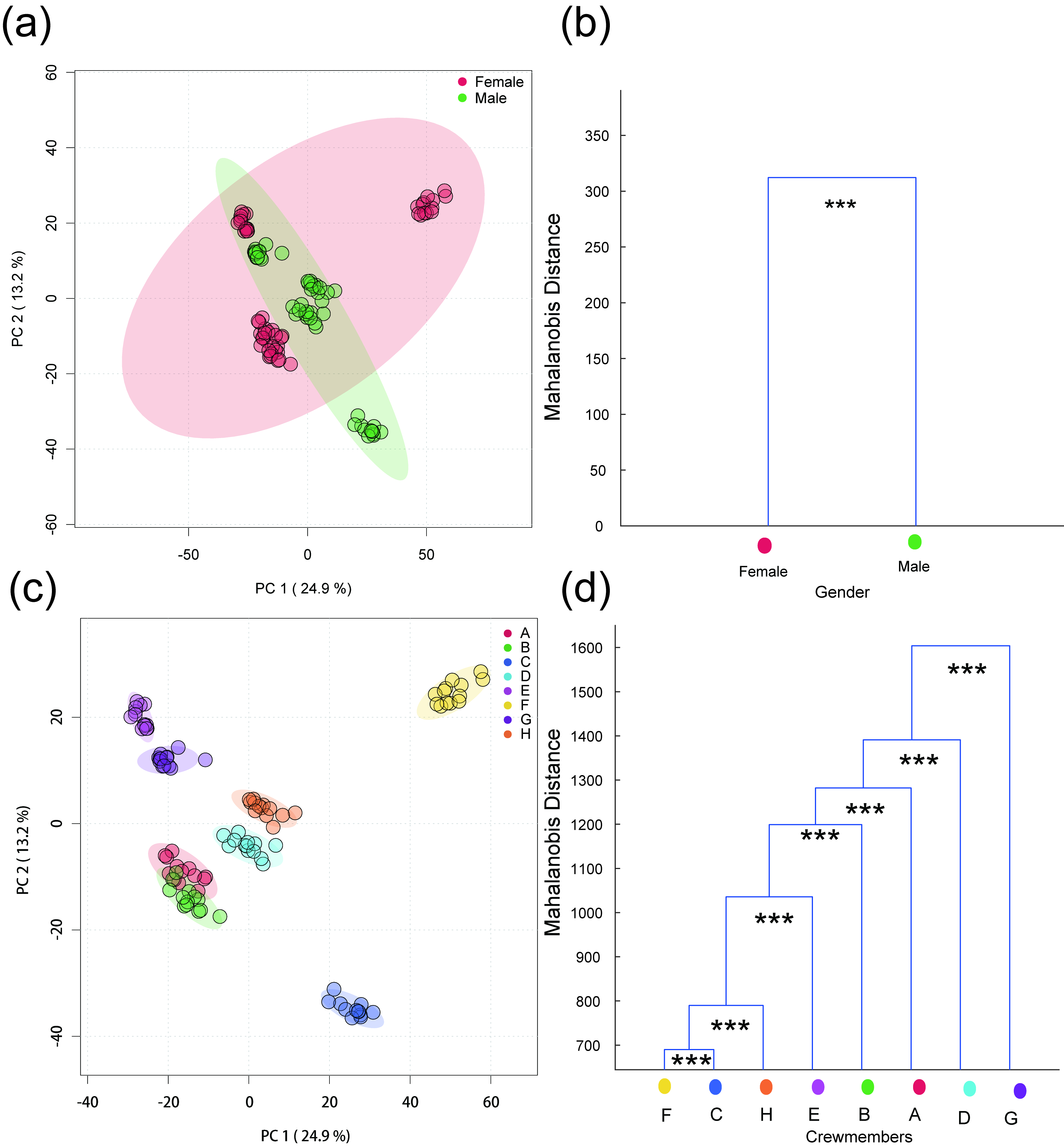

Supplement: Supplementary file 3 — Additional file 2: Fig. S1. The composition of gut microbiota had significant individual and gender differences. (a, c) PCA scores plots based on the relative abundance of gut microbiota at the species level in different individuals and genders. (b, d) Clustering of different groups based on mahalanobis distances calculated using MANOVA, *** P< 0.001. [file 40168_2023_1506_MOESM2_ESM.tif]

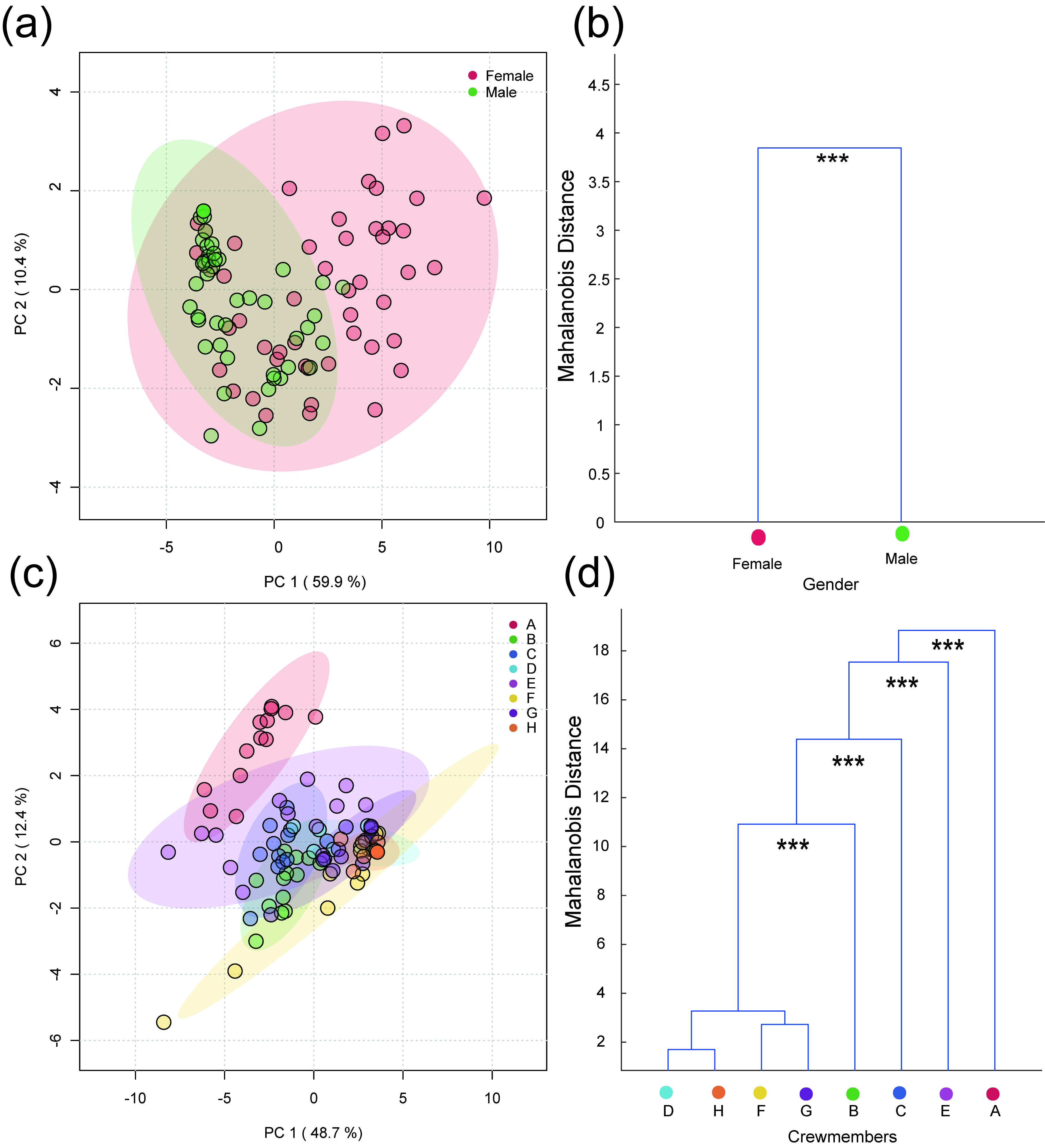

Supplement: Supplementary file 4 — Additional file 3: Fig. S2. The scores of psychological factors had significant individual and gender differences. (a, c) PCA scores plots based on the scores of psychological factors in different individuals and genders. (b, d) Clustering of different groups based on mahalanobis distances calculated using MANOVA, *** P< 0.001. [file 40168_2023_1506_MOESM3_ESM.tif]

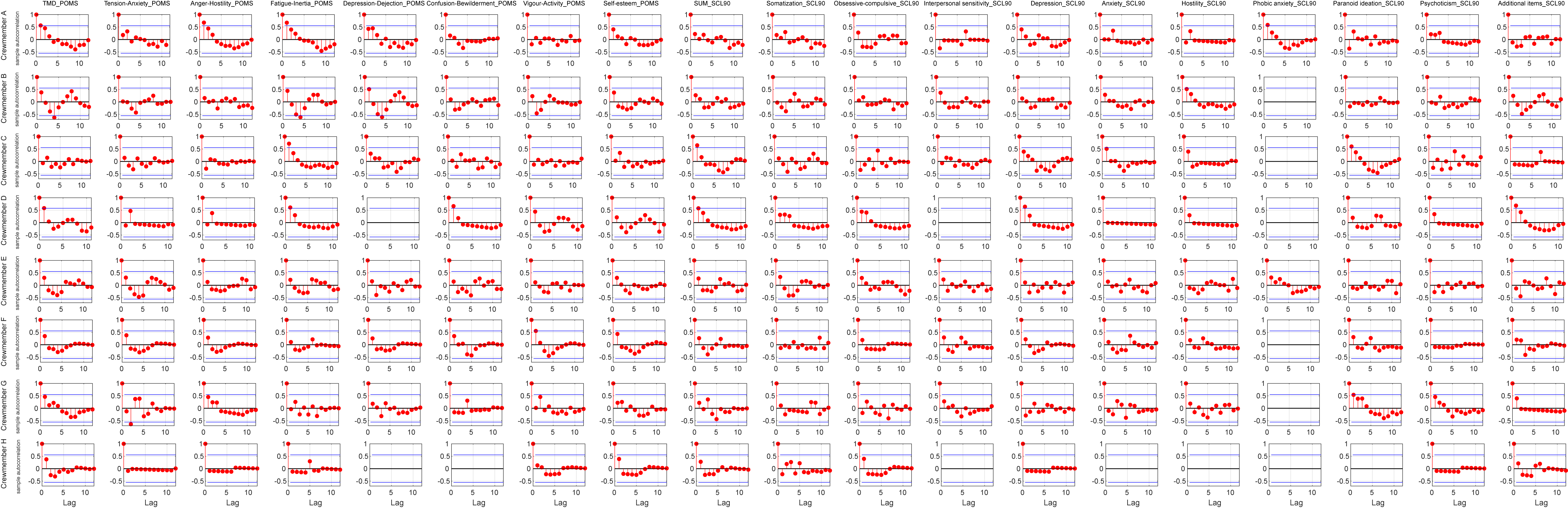

Supplement: Supplementary file 5 — Additional file 4: Fig. S3. The sample autocorrelation function plots (ACF) of the scores variation of each psychological factor. Blue lines indicate upper and lower range of confidential region, sample autocorrelation that falls into the confidential region indicate stability of the mood. The results showed no significant autocorrelation and the crewmembers’ psychological variations were stationary stochastic process. [file 40168_2023_1506_MOESM4_ESM.tif]

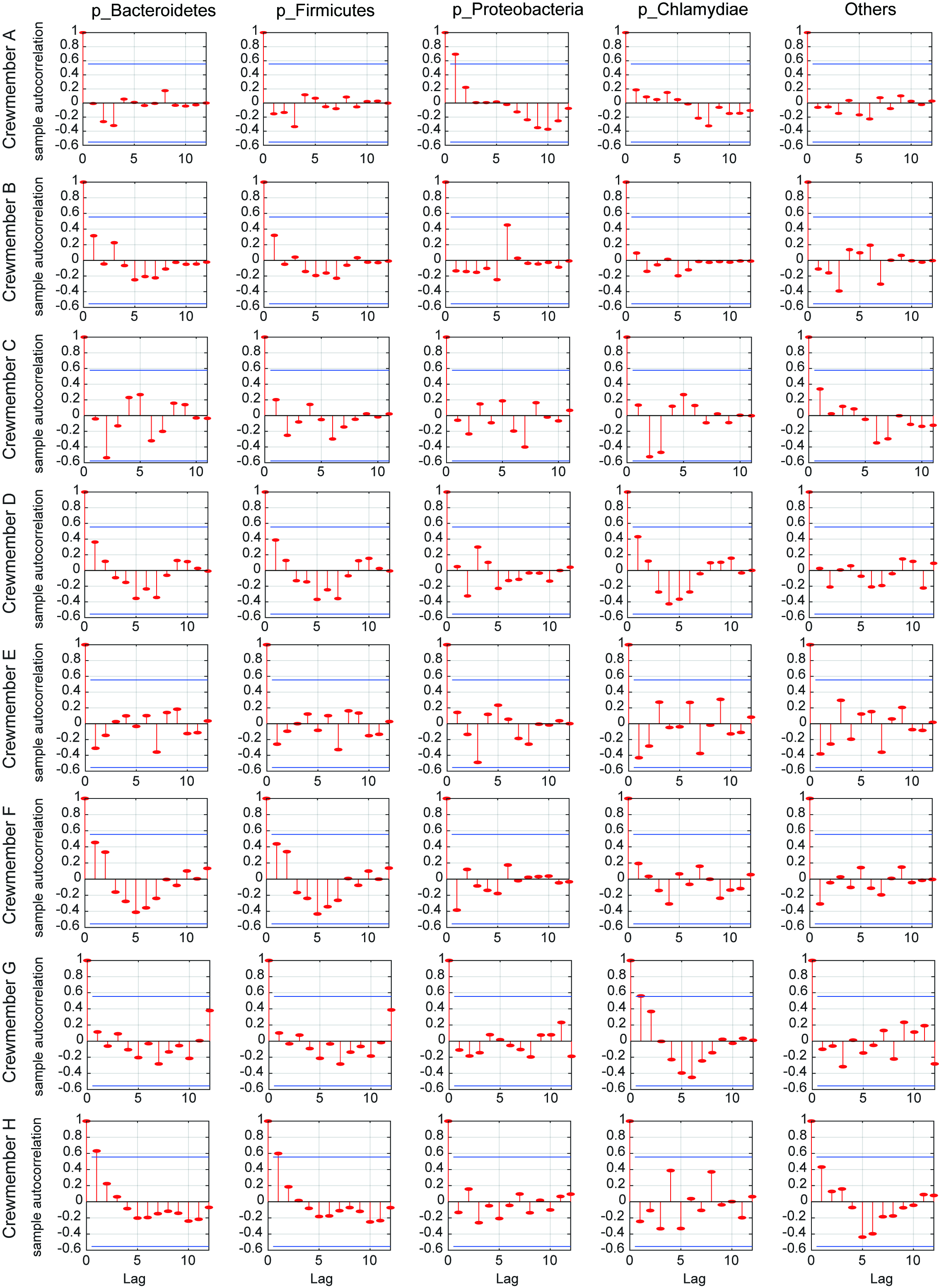

Supplement: Supplementary file 6 — Additional file 5: Fig. S4. The sample autocorrelation function plots (ACF) of the gut microbiome composition at the phylum level. Blue lines indicate upper and lower range of confidential region, sample autocorrelation that falls into the confidential region indicate stability of the changes of gut microbiota. The results showed no significant autocorrelation and the changes of gut microbiota with time was a static stochastic process. [file 40168_2023_1506_MOESM5_ESM.tif]

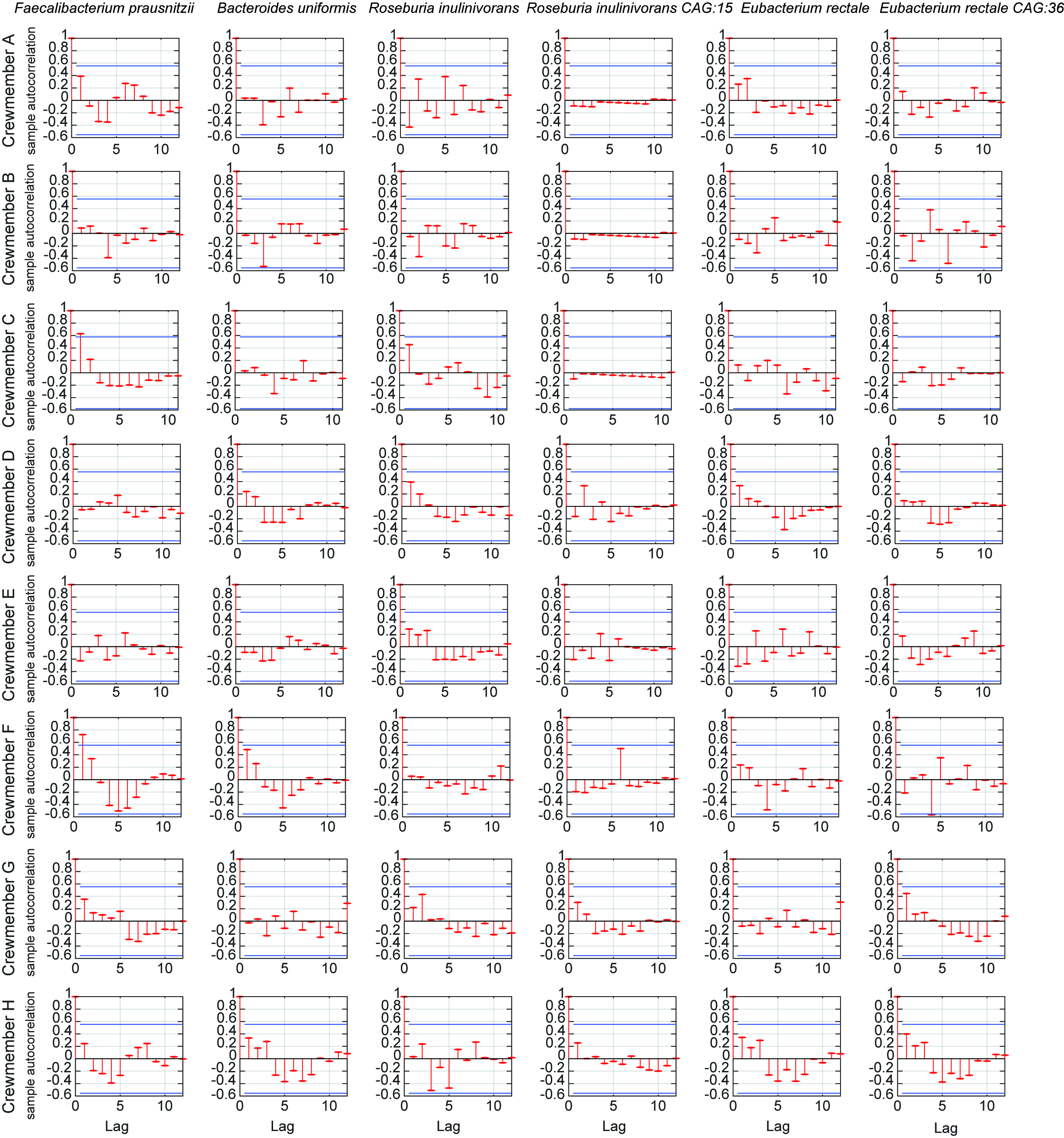

Supplement: Supplementary file 7 — Additional file 6: Fig. S5. The sample autocorrelation function plots (ACF) of the potential psychobiotics. Blue lines indicate upper and lower range of confidential region, sample autocorrelation that falls into the confidential region indicate stability of the changes of potential psychobiotics. The results showed no significant autocorrelation and the changes of potential psychobiotics with time was a static stochastic process. [file 40168_2023_1506_MOESM6_ESM.tif]

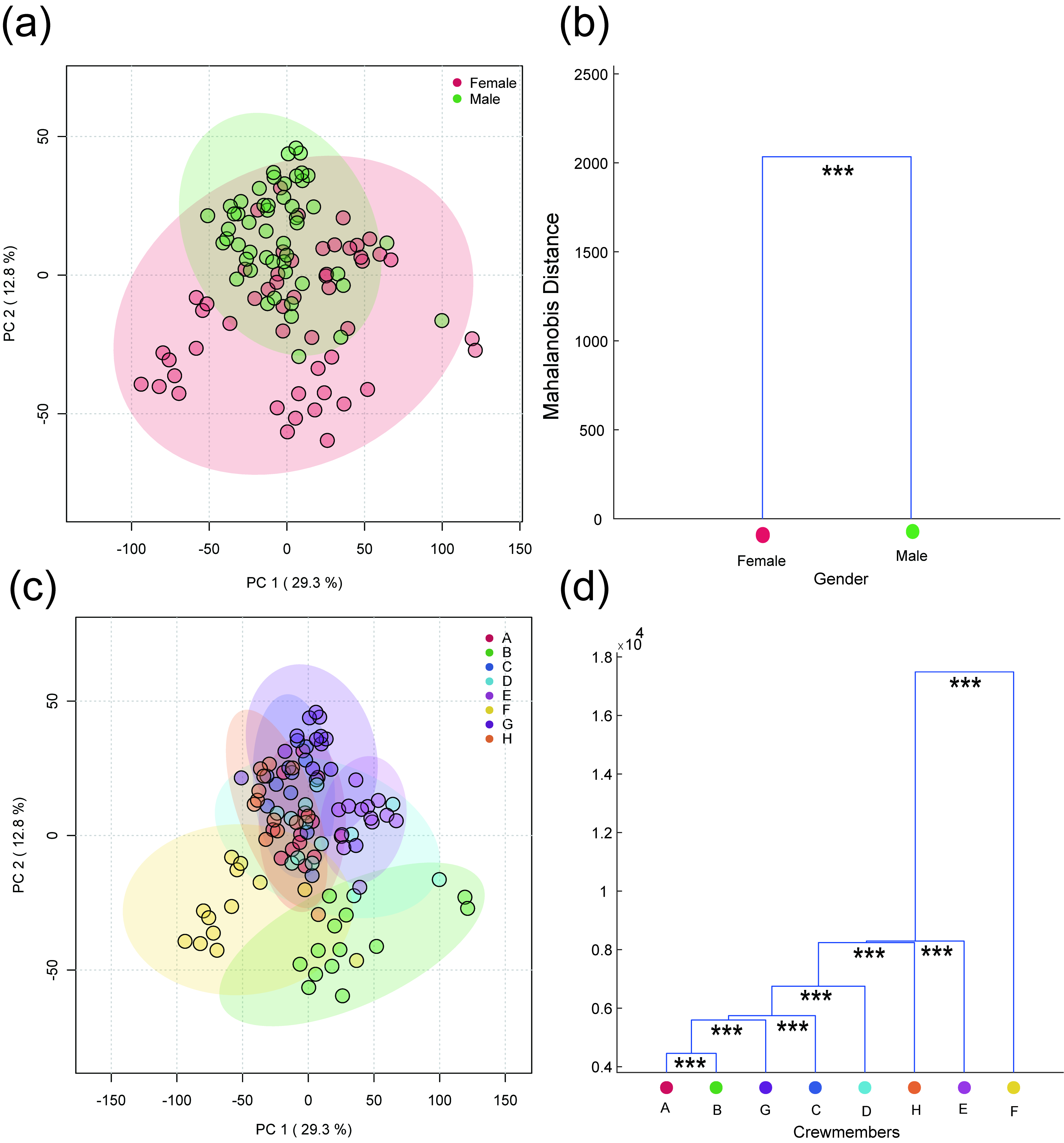

Supplement: Supplementary file 8 — Additional file 7: Fig. S6. The relative abundance of metagenomic function at the KO level had significant individual and gender differences. (a, c) PCA scores plots based on the relative abundance of metagenomic function at the KO level in different individuals and genders, respectively. (b, d) Clustering of different groups based on mahalanobis distances calculated using MANOVA, ***, P< 0.001. [file 40168_2023_1506_MOESM7_ESM.tif]

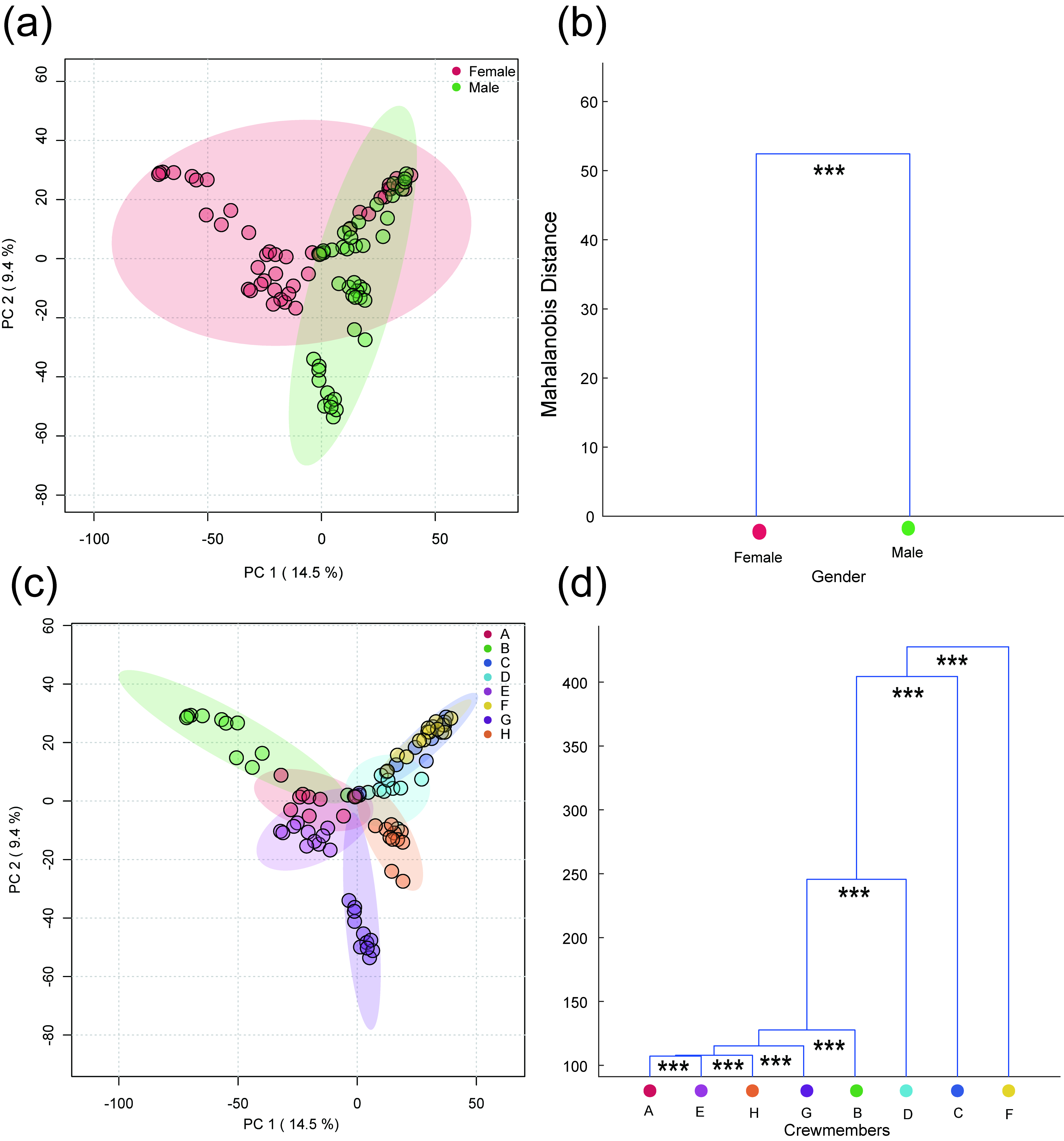

Supplement: Supplementary file 9 — Additional file 8: Fig. S7. The protein groups of the gut microbiota had significant individual and gender differences. (a, c) PCA scores plots based on the protein groups of the gut microbiota in different individuals and genders, respectively. (b, d) Clustering of different groups based on mahalanobis distances calculated using MANOVA, ***, P< 0.001. [file 40168_2023_1506_MOESM8_ESM.tif]

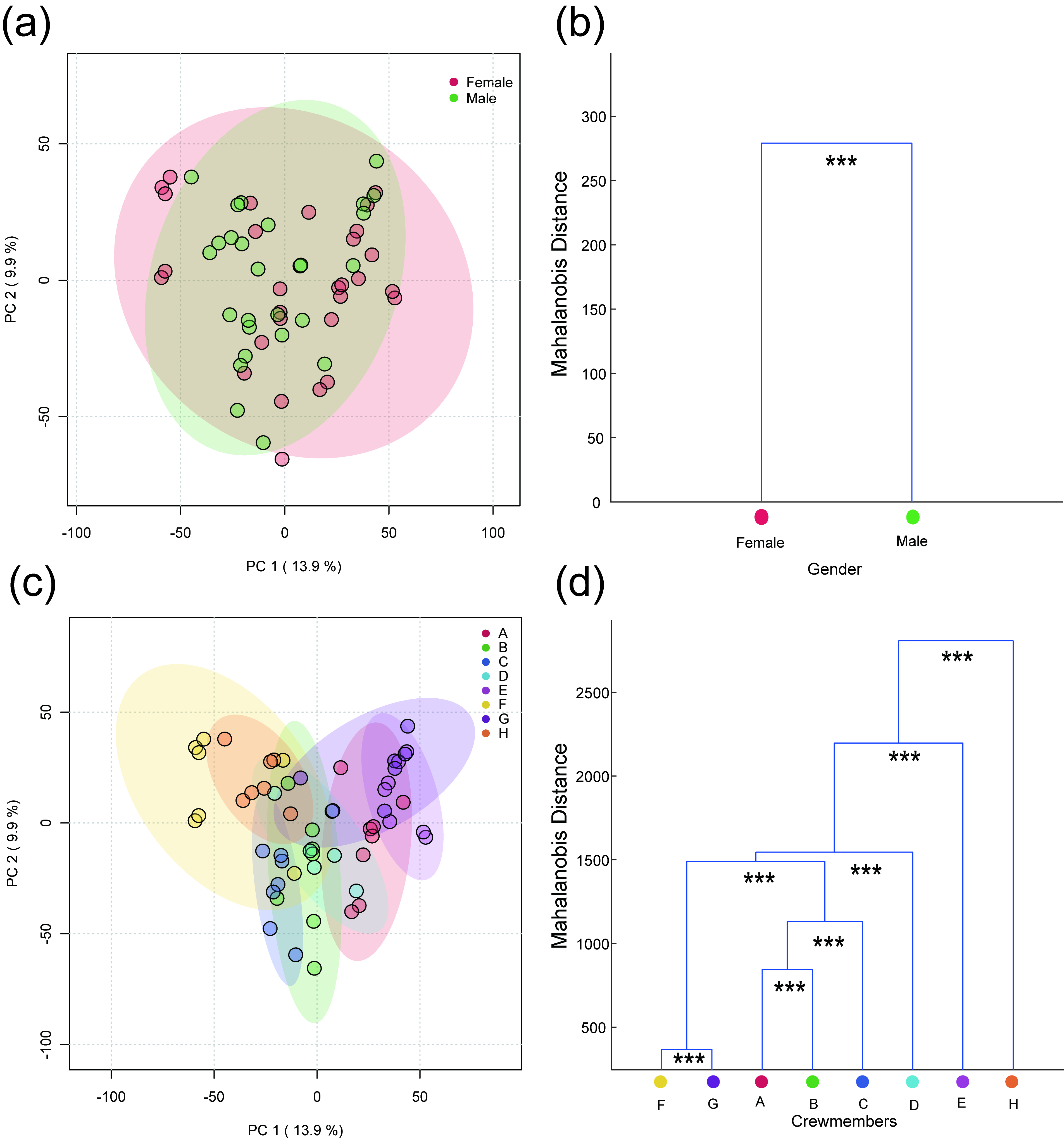

Supplement: Supplementary file 10 — Additional file 9: Fig. S8. The fecal metabolites in ES +/- had significant individual and gender differences. (a, c) PCA scores plots based on the fecal metabolites in ES +/- in different individuals and genders, respectively. (b, d) Clustering of different groups based on mahalanobis distances calculated using MANOVA, *** P< 0.001. [file 40168_2023_1506_MOESM9_ESM.tif]

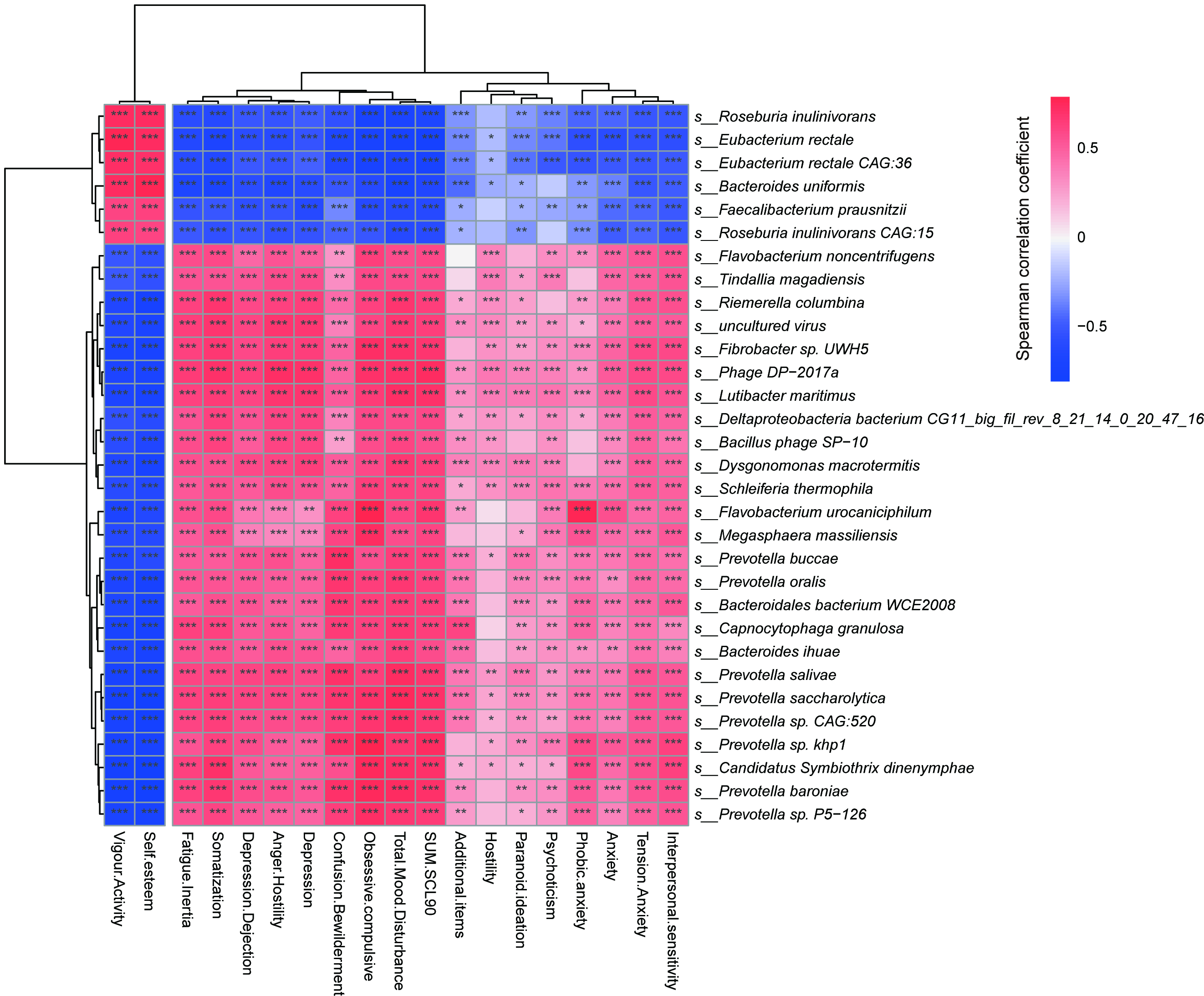

Supplement: Supplementary file 11 — Additional file 10: Fig. S9. Heatmap of Spearman’s correlation for potential psychobiotics and psychological factor scores. The species whose correlation coefficient |R| was ≥ 0.5 (P < 0.001) in more than 50% of the psychological factors are shown here. The scaling of correlation coefficient is represented by color depth—a positive correlation is expressed in red and a negative correlation in blue. *P ≤ 0.05, **P ≤ 0.01, ***P ≤ 0.001. [file 40168_2023_1506_MOESM10_ESM.tif]

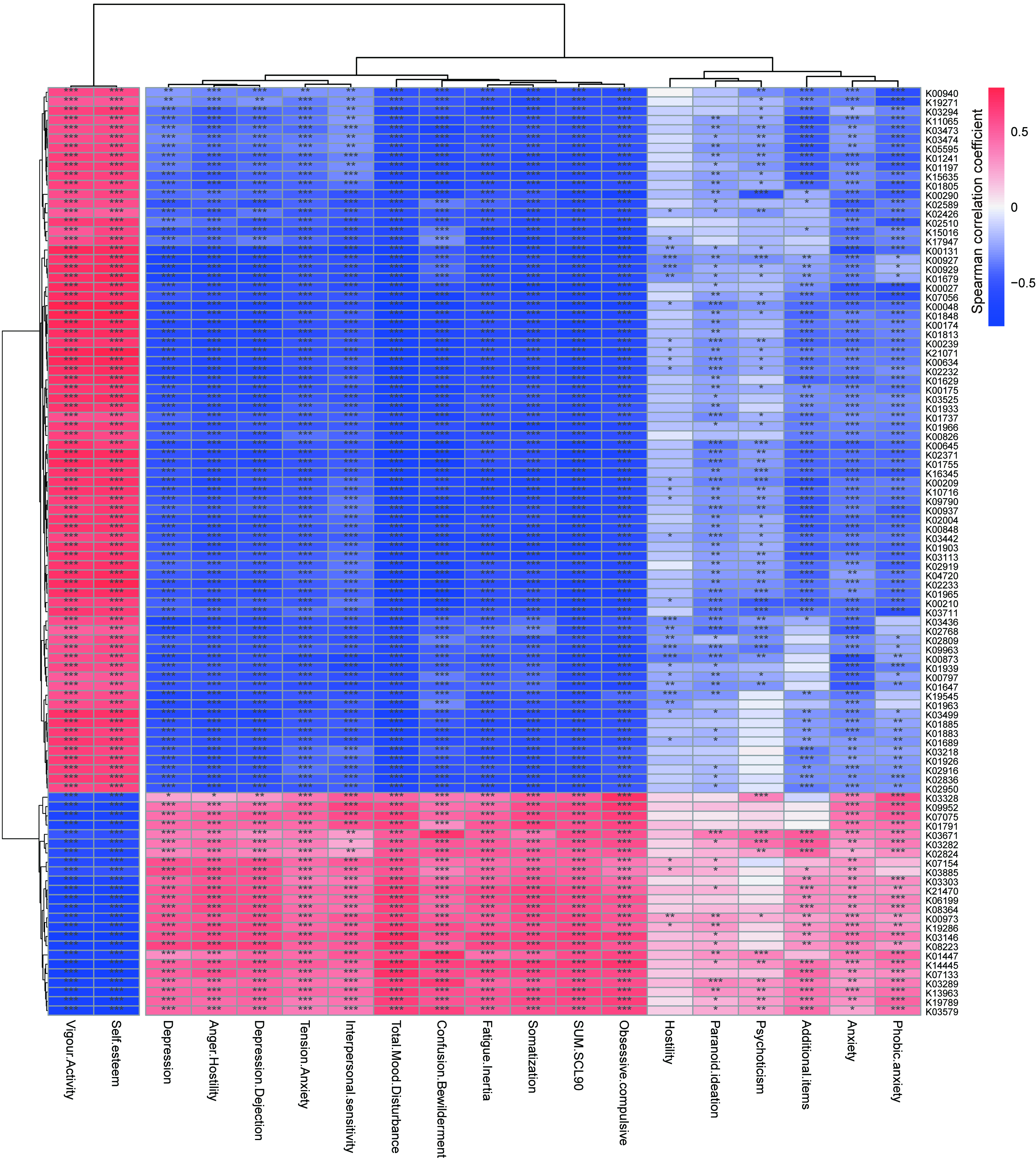

Supplement: Supplementary file 12 — Additional file 11: Fig. S10. Heatmap of Spearman’s correlation for key Kyoto Encyclopedia of Genes and Genomes ortholog groups (KOs) and psychological factor scores. The KOs whose correlation coefficient |R| was ≥0.5 (P < 0.001) in top 100 of the psychological factors are shown here. The scaling of correlation coefficient is represented by color depth—a positive correlation is expressed in red and a negative correlation in blue. *P ≤ 0.05, **P ≤ 0.01, ***P ≤ 0.001. [file 40168_2023_1506_MOESM11_ESM.tif]

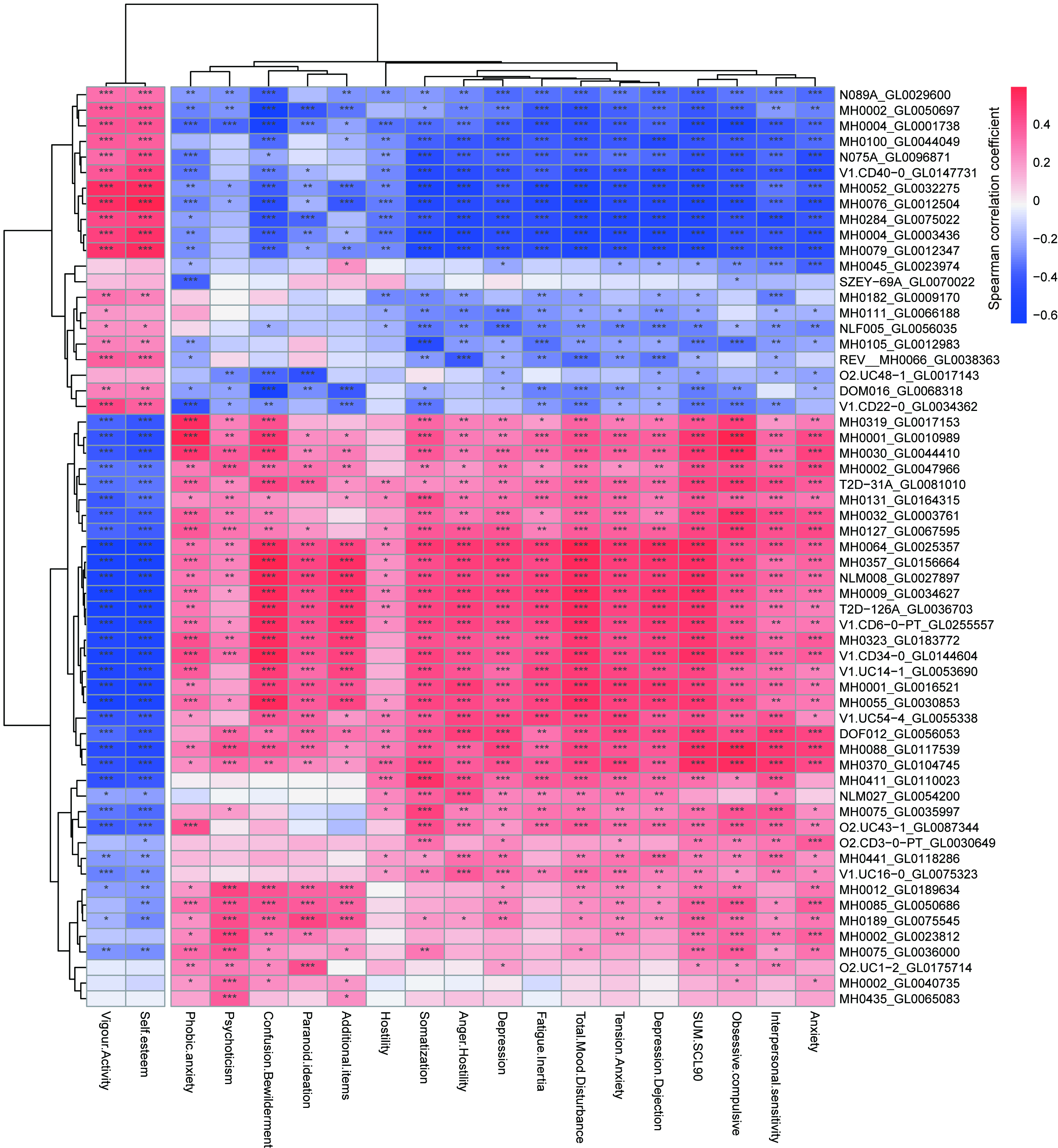

Supplement: Supplementary file 13 — Additional file 12: Fig. S11. Heatmap of Spearman’s correlation for key protein groups and psychological factor scores. The protein groups whose correlation coefficient |R| was ≥0.35 (P<0.001) in top 60 of the psychological factors are shown here. The scaling of correlation coefficient is represented by color depth—a positive correlation is expressed in red and a negative correlation in blue. *P ≤ 0.05, **P ≤ 0.01, ***P ≤ 0.001. [file 40168_2023_1506_MOESM12_ESM.tif]
